# Transformation Asymmetry and the Evolution of the Bacterial Accessory Genome

**DOI:** 10.1093/molbev/msx309

**Published:** 2017-12-01

**Authors:** Katinka J Apagyi, Christophe Fraser, Nicholas J Croucher

**Affiliations:** 1 MRC Centre for Outbreak Analysis and Modelling, Department of Infectious Disease Epidemiology, Imperial College London, London, United Kingdom; 2 Big Data Institute, Nuffield Department of Medicine, University of Oxford, Oxford, United Kingdom

**Keywords:** recombination, bacterial evolution, mobile elements, horizontal gene transfer, transformation, pneumococcus

## Abstract

Bacterial transformation can insert or delete genomic islands (GIs), depending on the donor and recipient genotypes, if an homologous recombination spans the GI’s integration site and includes sufficiently long flanking homologous arms. Combining mathematical models of recombination with experiments using pneumococci found GI insertion rates declined geometrically with the GI’s size. The decrease in acquisition frequency with length (1.08×10^−3 ^bp^−1^) was higher than a previous estimate of the analogous rate at which core genome recombinations terminated. Although most efficient for shorter GIs, transformation-mediated deletion frequencies did not vary consistently with GI length, with removal of 10-kb GIs ∼50% as efficient as acquisition of base substitutions. Fragments of 2 kb, typical of transformation event sizes, could drive all these deletions independent of island length. The strong asymmetry of transformation, and its capacity to efficiently remove GIs, suggests nonmobile accessory loci will decline in frequency without preservation by selection.

## Introduction

Acquisition of genomic islands (GIs) by bacteria can result in increased virulence (Groisman and Ochman 1996), antibiotic resistance (Dobrindt et al. 2004), or evasion of vaccine-induced immunity (Croucher et al. 2015). Such additions may be driven by the GIs themselves if they are mobile genetic elements (MGEs). Consequently, evolutionary models of the bacterial accessory genome have tended to focus on the gain of novel loci, which either add into the existing genome (Baumdicker et al. 2012; Collins and Higgs 2012) or displace recipient genes (Haegeman and Weitz 2012; Lobkovsky et al. 2013). To maintain stable genome sizes (Mira et al. 2001; Dagan and Martin 2007), some models impose a fitness cost on this expansion (Marttinen et al. 2015), attributed to selection against the energetic costs of DNA replication (Hogg et al. 2007; Baumdicker et al. 2012). Typically, gene loss is modeled as spontaneous deletion (Vogan and Higgs 2011; Baumdicker et al. 2012; Collins and Higgs 2012), reflecting the mutational bias (Mira et al. 2001) that deletions appear to be both larger and more frequent than insertions (Kuo and Ochman 2009).

The acquisition of non-MGE GIs typically requires homologous recombination between similar sequences, shared by the donor and recipient, flanking the GI. This can occur through natural transformation, the import of exogenous DNA by the competence machinery. Although the evolutionary advantage of transformation remains controversial, such import of novel genes has been proposed as a possible benefit of the competence machinery (Hogg et al. 2007; Johnston et al. 2013). Transformation also has the potential to remove GIs by replacing them with DNA from a donor that lacks the island. Despite rarely featuring in evolutionary models, this process may be advantageous if it removes deleterious GIs, such as parasitic MGEs (Croucher et al. 2016). Such RecA-mediated recombination is expected to seamlessly stitch the flanking regions together (Rosselli and Stasiak 1991; Johnston et al. 2013), without the costs associated with spontaneous deletion, such as damaging surrounding regions or leaving behind nonfunctional GI fragments.

Two criteria must be met for deletion of GIs, particularly MGEs, by transformation to be biologically relevant. First, transformation must exhibit a pronounced asymmetry toward deleting, rather than inserting, heterologous sequences. Second, deletions of single genes and 10- to 30-kb GIs must occur with similar efficiency. Preferential deletion, rather than import, of sequence by transformation has been previously observed in *Streptococcus pneumoniae* (Claverys et al. 1980; Lefevre et al. 1989) and *Bacillus subtilis* (Adams 1972), although more recently contradictory results have been recorded (Pasta and Sicard 1996). However, the mutations transferred in these studies were small, else their size was not precisely established, hence their relevance to typical GIs is uncertain (Croucher et al. 2014). Here, we quantify the asymmetry and efficiency with which transformation eliminates GIs from chromosomes.

## Results

### Assaying the Properties of Transformation

**Fig. 1:**
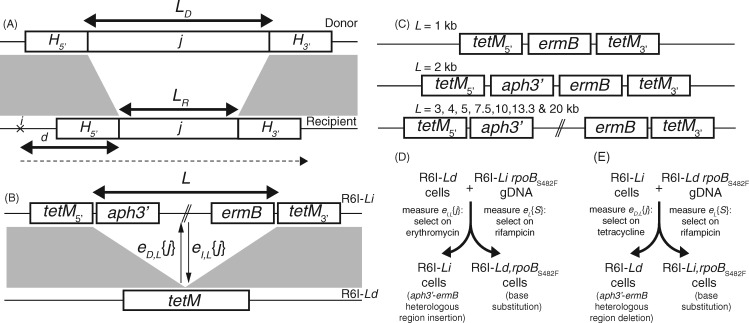
Exchange of heterologous regions through homologous recombination. (*A*) Description of the recombination process. Donor and recipient DNA sequences are shown with gray bands linking regions of sequence similarity, separated by a central heterologous locus *j*, of length *L*_D_ in the donor and *L*_R_ in the recipient. The minimum lengths of the flanking homologous arms necessary for exchange through homologous recombination, *H_5′_* and *H_3′_*, are shown on either side. The dashed line indicates an homologous recombination initiating at position *i, d* bases upstream of *j*. (*B*) Genotypes used in the experimental system, displayed as in (*A*). The R6I*-Li* genotype had either *ermB*, or *ermB* and *aph3′*, inserted between two halves of the *tetM* gene. The R6I*-Ld* genotype had an intact *tetM* gene, with all intervening sequence in the complementary R6I*-Li* genotype removed. Rifampicin-resistant derivatives of both were generated through transformation with an *rpoB* allele encoding an S482F substitution. (*C*) Structure of the R6I-*Li* genotypes. For *L* = 1 kb, the insert was a single *ermB* gene; for *L* = 2 kb, both *ermB* and *aph3′* were inserted within *tetM*; and for *L* ≥ 3 kb, these genes flanked nonessential DNA to generate constructs with the specified lengths. (*D*) Assaying insertion of heterology through transformation. Each R6I*-Ld* genotype was transformed with genomic DNA from the complementary R6I*-Li rpoB*_S482F_ genotype. Insertion of heterology (*e_I,L_*{*j*}) was inferred from counting erythromycin-resistant colonies, and acquisition of SNPs (*e_L_*{*S*}) was inferred from counting rifampicin-resistant colonies. (*E*) Assaying deletion of heterology through transformation. Each R6I*-Li* genotype was transformed with genomic DNA from the complementary R6I*-Ld rpoB*_S482F_ genotype. Deletion of heterology (*e_D,L_*{*j*}) was inferred from counting tetracycline-resistant colonies, and acquisition of SNPs (*e_L_*{*S*}) was inferred from counting rifampicin-resistant colonies.

Figure 1*A* describes the components of a model of GI exchange through homologous recombination. A GI, *j*, of length *L*_D_ in the donor DNA and *L*_R_ in the recipient cell is exchanged between cells, *e*{*j*}, if a recombination initiating at *i* (a distance *d* from *j*) spans not just *j* but also the minimum lengths for homologous arms, *H*_5__′_ and *H*_3__′_, on both sides. Assuming homologous recombinations have a geometric length distribution (Croucher et al. 2012), the probability of GI transfer relative to the exchange of a single-nucleotide polymorphism (SNP) *S*, *e*{*S*} can be quantified as (supplementary text S1, Supplementary Material online):

$$\frac{p(e\{j\})}{p(e\{S\})}=\;{\tau_{I}\left(1-\lambda_{I}\right)}^{L}$$

Where λ_I_ is the per-base pair rate at which recombinations terminate in heterologous regions, and the factor τ_I_ accounts for length-independent differences between the efficiency of GI and SNP transformation. The rate of GI exchange depends on a length, *L*; yet how *L* relates to *L*_D_ and *L*_R_ results in four models with distinct evolutionary implications (supplementary text S1, Supplementary Material online). In two models, transformation is symmetrical: if any heterology between the donor and recipient DNA inhibits recombination (“heterology limited” model; *L *=* L*_D_ + *L*_R_), then large GI insertion and deletion will be slow, whereas if GI movement is limited only by homologous arm dynamics, all sizes will exchange at the same rate (“annealing limited” model; *L* = 0). In two of these models, transformation is asymmetrical: GI insertion may be more efficient than deletion if transformation is limited by its size in the recipient genome (“deletion limited” model; *L *=* L*_R_), else if exchange is limited by its size in the donor DNA, deletion may be more efficient than insertion (“insertion limited” model; *L *=* L*_D_).

To test these models, an experimental system was generated to measure GI exchange (*e*{*j*}) relative to *L*_R_ and *L*_D_. The unencapsulated strain *Streptococcus pneumoniae* R6x (Tiraby and Fox 1973) was modified through a streptomycin resistance mutation (rpsL*), insertion of the integrative and conjugative element ICE*Sp*23FST81 at *att_rplL_* (Croucher et al. 2009), and deletion of the phase variable *ivr* restriction-modification locus (Croucher et al. 2014) (supplementary fig. S1, Supplementary Material online). The use of this R6x rpsL* Δ*ivr att_rplL_*::[ICE*Sp*23FST81] genotype, henceforth named R6I, as a background for both donors and recipients meant transformations would not be inhibited by mismatch repair (Tiraby and Fox 1973), capsule (Yother et al. 1986), divergence between orthologous sequences (Majewski et al. 2000) or restriction endonucleases (Johnston et al. 2013). To assay the relative rates at which GIs of length *L* were inserted (*e_I,L_*{*j*}) and deleted (*e_D,L_*{*j*}) through transformation, four genotypes were constructed for each of nine tested *L* values (fig. 1). The first, R6I*-Li*, had *L* kb of sequence within ICE*Sp*23FST81 separating the 5′ half of the *tetM* tetracycline resistance gene, immediately upstream of an introduced *aph3′* aminoglycoside resistance gene, from the 3′ half of *tetM*, immediately downstream of an introduced *ermB* erythromycin resistance gene (fig. 1*B*). The exception was *L *= 1 kb, where the *tetM* gene halves were separated by only *ermB* (fig. 1*C*). The second, R6I*-Ld*, had an intact *tetM* gene, the intervening *L* kb of sequence having been removed, including the *aph3′* and *ermB* genes. PCR assays verified these genotypes had undergone the expected changes (supplementary figs. S2 and S3, Supplementary Material online). The third and fourth genotypes, R6I*-Li rpoB*_S482F_ and R6I*-Ld rpoB*_S482F_, were rifampicin-resistant variants of the first two generated through transformation with an *rpoB* allele encoding an S482F substitution (supplementary figs. S5 and S6, Supplementary Material online).

Genomic DNA (gDNA) exhibiting little evidence of degradation (supplementary fig. S7, Supplementary Material online) from R6I*-Li rpoB*_S482F_ was used to transform R6I*-Ld* cells (fig. 1*D*), followed by selection on rifampicin plates, to measure *e_L_*{*S*}, and erythromycin plates, to measure *e_I,L_*{*j*}. Spontaneous emergence of rifampicin resistance, which would distort *e_L_*{*S*}, was infrequent (supplementary fig. S8, Supplementary Material online). Similarly, PCR assays confirmed erythromycin selection was specific in identifying recombinants that had acquired the full heterologous locus (supplementary fig. S9, Supplementary Material online). Conversely, transformation of R6I*-Li* cells with R6I*-Ld rpoB*_S482F_ gDNA was followed by selection on rifampicin plates, to measure *e_L_*{*S*}, and tetracycline plates, to measure *e_D,L_*{*j*} (fig. 1*E*). PCR amplification again confirmed selection on tetracycline was specific for the deletion of all the intervening sequence (supplementary fig. S10, Supplementary Material online).

### Transformation Is Asymmetric and Insertion Limited

The analysis encompassed 183 biological replicates, with at least six per recipient genotype, each of which was estimated to generate at least 250 rifampicin-resistant transformants. The data showed clear variation in *e_L_*{*S*} between the constructed genotypes (supplementary fig. S11, Supplementary Material online), which could not be attributed to differences in growth rates (supplementary fig. S12, Supplementary Material online), and therefore an altered model was jointly fitted across all experimental results through maximum likelihood (supplementary text S1, Supplementary Material online):

$$\frac{p(e_{L}\{j\})}{p(e_{L}\{S\})}=\;{\tau_{I}\tau_{g}\left(1-\lambda_{I}\right)}^{L}$$

$$p\left(e_{L}\left\{S\right\}\right)=\tau_{g}$$

Where τ_g_ represented a genotype-specific transformation rate, whereas τ_I_ (the length-independent relative GI transformation rate) and λ_I_ (the per-base pair rate of recombination termination in *j*) were fixed across all genotypes (supplementary fig. S11, Supplementary Material online). The experiments measuring *e_L_*{*j*} found a geometric decline with *L*, consistent with the “insertion limited” and “heterology limited” models (*L* α *L_D_*). Rare insertions were observed at *L* = 20 kb only with an elevated concentration of donor DNA. Using bootstrapping to calculate the confidence intervals, τ_I_ was estimated as 3.49 (full bootstrap range: 0.98–6.41), and λ_I_ was estimated as 1.08×10^−3 ^bp^−1^ (bootstrap range: 6.81×10^−4^–1.31×10^−3 ^bp^−1^). Transformation is therefore inefficient at inserting long GIs.

**Fig. 2:**
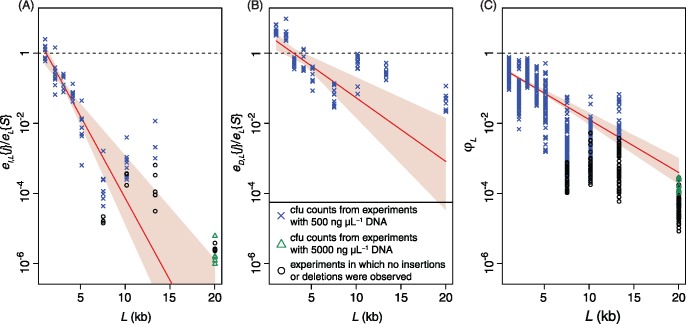
Rates of polymorphism transfer through transformation. (*A*) Relationship between heterologous locus length, *L*, and rate of insertion, *e_I,L_*{*j*}, relative to rate of SNP acquisition, *e_L_*{*S*}. Each point represents a biological replicate. Blue crosses and green triangles represent *e_I,L_*{*j*} estimates from counting colonies following transformation with 500 ng, or 5,000 ng, donor DNA, respectively; black circles represent experiments where *e_I,L_*{*j*} was too low to be experimentally detectable following transformation, hence a value of *e_I,L_*{*j*} = 0.5 cfu ml^−1^ was assumed for display purposes. The red line displays the best fitting relationship of the form τ_*I*_(1−λ_I_)^*L*^. The pink shaded region indicates the full range of associated uncertainty inferred from 100 bootstrap replicates. The horizontal dashed line indicates where *e_I,L_*{*j*} equals *e_L_*{*S*}. (*B*) Relationship between heterologous locus length, *L*, and rate of deletion, *e_D,L_*{*j*}, relative to rate of SNP acquisition, *e_L_*{*S*}. Results are displayed as in panel (*A*). (*C*) Asymmetry of transformation, ϕ_*L*_. The points represent every ratio of *e_I,L_*{*j*}/*e_L_*{*S*} to *e_D,L_*{*j*}/*e_L_*{*S*} for each *L*. The characters correspond to those of the underlying *e_I,L_*{*j*}/*e_L_*{*S*} value in panel (*A*); *e_I,L_*{*j*} values of zero are again substituted for 0.5 cfu ml^−1^. The red line and pink shaded region display the best-fitting relationship of the form ϕ_*0*_(1−λ_ϕ_)^*L*^, and the associated uncertainty inferred from 100 bootstrap replicates.

To distinguish between the “insertion limited” and “heterology limited” models, the effect of *L*_R_ on the rate of transformation-mediated deletion was measured for each *L*. Consistent with the latter model, the deletion frequency *e_D,L_*{*j*} was highest for *L* ≤ 2 kb (fig. 2*B*). Fitting the geometric decline model estimated τ_I_ as 3.51 (bootstrap range: 1.70–6.44), and λ_I_ as 4.18×10^−4 ^bp^−1^ (bootstrap range: 2.69×10^−4^–6.05×10^−4 ^bp^−1^). However, for *L* ≥ 3 kb, *e_D,L_*{*j*} varied by recipient genotype rather than *L*, more consistent with the “insertion limited” model. Even at *L* = 10 kb, *e_D,L_*{*j*} was ∼50% of *e_L_*{*S*}. Hence transformation-mediated deletion of GIs is substantially more efficient than their insertion.

The asymmetry statistic ϕ_*L*_, quantifying the relative insertion and deletion rates for a GI of length *L*, was calculated as (fig. 2*C*):

$$\phi_{L}=\frac{e_{I,L}je_{D,L}\{S\}}{e_{I,L}\left\{S\right\}e_{D,L}\{j\}}$$

The relationship between ϕ_*L*_ and *L* suggested the model:

$$\phi_{L}={\phi_{0}\left(1-\lambda_{\phi }\right)}^{L}$$

A maximum likelihood fit estimated ϕ_0_, the asymmetry associated with a minimally sized GI, as 0.413 (bootstrap range: 0.355–0.470), and the parameter determining the rate of change with *L*, λ_ϕ_, as 3.47×10^−4 ^bp^−1^ (bootstrap range: 2.99×10^−4^–3.92×10^−4 ^bp^−1^). Hence transformation is highly asymmetric, favoring deletions across all *L*.

### Homologous Arm Lengths Unaffected by Size of Deletion

**Fig. 3:**
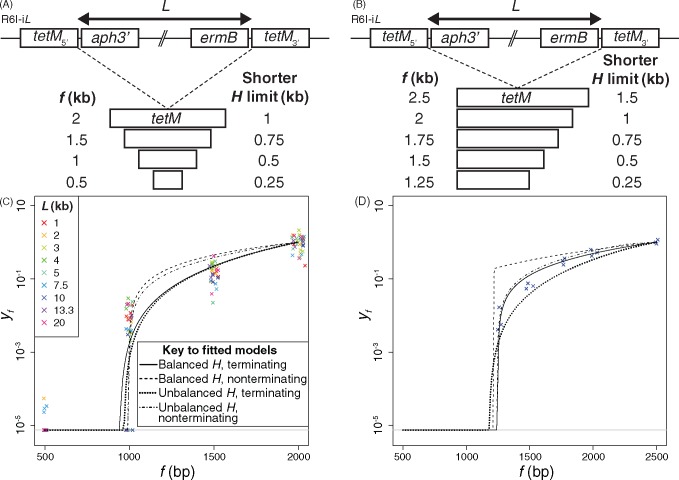
Characterizing the length distribution of homologous arms. (*A*) Deletion of loci with different *L* by *tetM* fragments of different lengths, *f*. Each fragment had similarity to an equal length of sequence on both flanks of the heterologous locus. (*B*) Efficiency of deletion with symmetrical homologous arms. The metric *y_f_* corresponds to the ratio *e_D,f_*{*j*}/*e_f_*{*S*} for a fragment of length *f* standardized to the mean of the same metric for the largest fragment (2 kb), adjusted to account for the differing number of DNA molecules available for transformation (described in supplementary text S2, Supplementary Material online). Hence across all *L*, the mean relative efficiency is one at *f* = 2 kb. Shorter fragments drove deletions less efficiently, hence were associated with *y_f_* < 1. Three biological replicates are shown for each genotype at each *f*, coloured according to the genotype of the recipient, which is of the type R6I-*Li*. The points for each value of *f* are distributed over a small fraction of the horizontal axis for display purposes. The horizontal gray line at the bottom represents the threshold to which all zero values were adjusted for plotting, and the curves show the fit of four models (see key). (*C*) Deletion of a region of heterology in R6I-10*d* by *tetM* fragments matching different lengths downstream of the heterologous locus (supplementary text S3, Supplementary Material online). Each fragment was identical to the 1 kb of *tetM* upstream of the heterologous locus, with different lengths matching the downstream region. (*D*) Efficiency of deletion with unbalanced homologous arms. Data are plotted as in panel (*B*), but only for R6I-10*d*. The *y_f_* metric is calculated in the same way, except the maximum *f* in this experiment is 2.5 kb, hence this is the point at which the mean *y_f_* is one.

The assay was modified to test whether the variation in deletion efficiency reflected length differences in the associated homologous arms. Each of the R6I*-Li* genotypes was simultaneously transformed with a *tetM* fragment of length *f*, which symmetrically spanned *j*, to measure *e_D,f_*{*j*} as counts of tetracycline-resistant transformants, and gDNA containing the *rpoB*_S482F_ allele, to measure *e_f_*{*S*} as counts of rifampicin-resistant transformants (fig. 3*A* and supplementary fig. S11, Supplementary Material online). The results for each genotype are shown in terms of the standardized rate of deletion, *e_D,f_*{*j*}/*e_f_*{*S*}, for multiple fragment sizes relative to the mean standardized rate with the maximally sized fragment *M*, *ē_D,M_*{*j*}/*ē_M_*{*S*}. This metric, *y_f_*, was calculated as:

$$y_{f}=\frac{e_{D,f}j{\overset{-}{e}}_{M}\{S\}f}{e_{D,f}\left\{S\right\}{\overset{-}{e}}_{M}\{j\}M}$$

The *f*/*M* ratio adjusts for the use of a fixed concentration of donor DNA, meaning the number of molecules available for transformation varies with fragment length. A reproducible increase in *y_f_* with *f* was observed (fig. 3*B*), with 500-bp fragments rarely causing deletions at a measureable rate. However, the consistency of the results between genotypes could not explain the irregular pattern of results in figure 2*B*.

Four different approaches were used to model the observed pattern of deletions (supplementary text S2 and fig. S14, Supplementary Material online), represented by the lines in figure 3*B*. The balanced models assumed homologous arms of at least *H* were necessary on each side of *j*, whereas the unbalanced models assumed the two homologous arms had to total 2 *H*, which could be unevenly spread across *j*. The other distinction related to whether the model required successful termination of recombination (terminating models), either through a randomly positioned nick in the donor DNA or other biochemical process, or assumed fragments were imported intact, and any recombination extending to the fragment’s end resolved there (nonterminating models). The four models estimated *H* as between 469 and 499 bp (supplementary table S1, Supplementary Material online), although it was difficult to identify the closest-fitting formulation. To distinguish between the hypotheses, this experiment was repeated with genotype R6I-10*d* and DNA fragments that asymmetrically spanned *j*, with one homologous arm constant and the other varying between 250 and 1,500 bp (fig. 3*C*). Only the unbalanced models estimated parameters similar to those from the first experiments, as deletions were consistently detectable when the variable homologous arm was just 250 bp (supplementary text S3, Supplementary Material online and fig. 3*D*). This demonstrates deletions can occur even with one foreshortened homologous arm, although the imperfect model fits suggest there are nevertheless some constraints on both homologous arm lengths. Deletion was relatively efficient even with small DNA fragments, with little increase in *y_f_* as *f* rose from 2 to 2.5 kb.

## Discussion

Transformation asymmetry is likely to have a strong impact on the evolution of the accessory genome, as recombinations of the mean size observed in the pneumococcus (∼2.3 kb) (Croucher et al. 2012) are able to efficiently delete 10- to 20-kb stretches of heterologous DNA, consistent with the size of pneumococcal GIs (Croucher et al. 2014). This assay should be conservative in estimating GI deletion efficiency, as mismatch repair would further inhibit the exchange of SNPs, but not GIs (Tiraby and Fox 1973); restriction-modification systems should inhibit GI acquisition, but not deletion (Johnston et al. 2013); and the deletions in this assay formed potentially deleterious artificial junctions and, in the case of R6I-20*i*, necessitated the loss of a putative toxin–antitoxin system (SPN23F12920-12930) (Dy et al. 2014). Although measured in a highly transformable laboratory-adapted strain, the estimate of λ_I_ from the decline of *e*{*j*} with *L* for insertions (1.08×10^−3 ^bp^−1^) was higher than a previous estimate of λ_R_, governing the exponential length distribution of core genome transformation events in a distinct clinical isolate (4.40×10^−4 ^bp^−1^) (Croucher et al. 2012). Hence the length of homologous recombinations and the spanning of heterologous regions may be limited by different mechanisms, such as RecA properties (Rosselli and Stasiak 1991) or donor DNA hydrolysis (Morrison and Guild 1972), else exhibit differing sensitivities to the same constraining process. Although these λ_I_ and λ_R_ estimates may be specific to pneumococcal transformation, similar principles will likely apply to all sequence exchange through RecA-mediated recombination, whether DNA is cut prior to packaging in a transducing phage or gene transfer agent (Lang et al. 2012), or imported from any potentially hydrolytic environment.

Therefore, the primary benefit of transformation seems more likely to be removal of deleterious GIs (Croucher et al. 2016), potentially counteracting MGE insertion through integrase-mediated recombination, than adaptation by GI acquisition (Hogg et al. 2007). Simulations of a recombining population using the asymmetry estimates suggest transformation would be effective at removing large MGEs, and even IS element insertions (Rocha 2016), given the results for *L* = 1 kb (supplementary fig. S15, Supplementary Material online). Alongside the observed mutational bias toward deletion (Mira et al. 2001; Kuo and Ochman 2009), this asymmetrical transfer of GIs suggests neutrally they should decline in frequency, congruent with the decay of genomes under relaxed selection (Kuo et al. 2009; Novichkov et al. 2009). Hence GIs surviving in transformable bacteria must either be advantageous to subpopulations through diversifying, frequency-dependent, or niche-specific selection, else evade elimination through elevated intercellular transmission.

## Materials and Methods

### Transformation Rate Assay

Generation of the DNA constructs and bacterial genotypes used in these experiments is described in supplementary text S4, Supplementary Material online. Each transformation assay used 1 ml of *S. pneumoniae* grown statically at 35 °C in Todd–Hewitt broth with 0.5% yeast extract (THY; Thermo Fisher Scientific) to an OD_600_ of 0.2–0.3. Five microliters of 500 mM calcium chloride (Sigma–Aldrich), 5 µl 5 ng µl^−1^ competence stimulating peptide 1, and 5 µl water containing 500 or 5,000 ng of genomic DNA, or 300 ng of PCR amplicon, were added. Transformants were selected on appropriately supplemented THY agar media (4 µg ml^−1^ rifampicin, 1 µg ml^−1^ erythromycin, or 10 µg ml^−1^ tetracycline) after 3 h of further incubation. Colonies were counted manually after 48 h.

### Statistical Analyses

The statistical models described in supplementary text S2, Supplementary Material online, were fitted to the data in figure 2 using maximum likelihood optimization with the Brent method in R (R Core Team 2017). Owing to the irregular outputs of the statistical models in supplementary text S3 and S4, Supplementary Material online, they were fitted to the data in figure 3 through least squares using simulated annealing in the “maxLik” package (Henningsen and Toomet 2011). 

## Supplementary Material

Supplementary data are available at *Molecular Biology and Evolution* online.

## Supplementary Material

Supplementary Data
