# Supplementary material for: Transformation Asymmetry and the Evolution of the Bacterial Accessory Genome
Source: Mol Biol Evol. 2017 Dec 1;35(3):575–81. doi: 10.1093/molbev/msx309 (PMC5850275; doi:10.1093/molbev/msx309)
Supplement: Supplementary Data [file msx309_supp.pdf]

Supplementary Materials

Transformation asymmetry and the evolution of the bacterial accessory genome

Text S1-S4
Figures S1-S15
Tables S1-S2
Supplementary References

Text S1: Models for transfer of heterologous sequence by homologous recombination

Assume a locus *j* of length L_D in the donor and L_R in the recipient that can be exchanged through recombination if the recombination includes both a homologous arm in the 5' region upstream of *j* that is of length at least $H_{5'}$, and a second homologous arm in the 3' region downstream of *j* that is of length at least $H_{3'}$ (Fig 1A). In this model, a balanced situation is assumed in which both $H_{5'}$ and $H_{3'}$ are of an identical length, *H*. The donor and recipient share a total core *C*, throughout which both have complete sequence identity, whereas it is assumed there is no sequence similarity within *j*. Recombinations are assumed to initiate at a uniform per nucleotide frequency of τ within *C*. The start point is denoted *i*, which is positioned *d* nucleotides upstream of *j* (Fig 1A). Recombinations that terminate within *j* are assumed to fail; only those which begin and terminate within *C*, and encompass *j*, $H_{5'}$ and $H_{3'}$ succeed in exchanging *j*, an event denoted *e*{*j*}. The probability of this, *p*(*e*{*j*}), given the initiation point *i*, can be expressed as:

$$p(e\{j\}|i) = p(r\{d\} \cap r\{j\} \cap r\{H_{3'}\}), \text{ for } H \leq d < C\text{-}H$$

Where notation of the type *r*{*d*} denotes a recombination spanning length *d*. Recombinations are assumed to terminate with a frequency of λ_R per base in regions of sequence similarity between the donor and recipient. They therefore have a geometric length distribution, in accordance with experimental and bioinformatic analyses (Croucher et al. 2012). Therefore the probability that a recombination spans a length of at least *d* can be expressed as:

$$p(r\{d\}) = (1 - \lambda_R)^d$$

Similarly, the homologous arm on the 3' side of *j* must span a region of sequence similarity, and so the probability that a recombination spans at least *H* bases on the 3' side of *j* is:

$$p(r\{H_{3'}\}) = (1-\lambda_R)^H$$

By analogy, if recombinations terminate with a frequency of λ_I per nucleotide in heterologous regions, then the probability that the recombination will span the locus *j*, defined as being divergent between the donor and recipient, with length *L*, is:

$$p(r\{j\}) = (1-\lambda_I)^L$$

The geometric distribution has the property that:

$$p(x > y+z) = p(x > y)p(x > z)$$

Therefore, returning to the expression for the exchange of locus *j*:

$$p(e\{j\}|i) = p(r\{d\} \cap r\{j\} \cap r\{H_{3'}\}), \text{ for } H \leq d < C\text{-}H$$

For a fixed initiation point *i*, this expression can be considered equivalent to the probability the recombination length, l_r, is equal to or greater than the combined total distance of *d*, *j* and $H_{3'}$. As each component of the probability is distributed geometrically, this overall probability can be expressed as:

$$p(e\{j\}|i) = p(l_r \geq d+L+H) = p(l_r \geq d)p(l_r \geq L)p(l_r \geq H)$$

Using the original terminology, this is equivalent to:

$$p(e\{j\} \mid i) = p(r\{d\})p(r\{L\})p(r\{H_{3'}\})$$

Therefore, combining the individual probabilities derived above:

$$p(e\{j\}| i) = (1-\lambda_R)^d(1-\lambda_I)^L(1-\lambda_R)^H \text{ for } H \leq d \leq C\text{-}H$$

$$p(e\{j\}| i) = (1-\lambda_R)^{d+H}(1-\lambda_I)^L \text{ for } H \geq d \geq C\text{-}H$$

To calculate the overall $p(e\{j\})$, it is necessary to sum over the possible range of d values on both the upstream and downstream sides of j, and adjust by the overall probability of any transformation initiating between the donor and recipient by including the per nucleotide rate of transformation event initiation, τ:

$$p(e\{j\}) = \sum_{d=H}^{C-H-1} 2\tau(1-\lambda_R)^{d+H}(1-\lambda_I)^L$$

This expression includes a factor of two to account for recombinations initiating on either side of j being able to exchange the locus, depending on the strand of the chromosome to which they anneal. Based on different assumptions about the nature of L, four different models with different consequences for bacterial evolution can be proposed:

1. Insertion-limited transformation: $L = L_D$, $\lambda_I > 0$

Under this model, L corresponds to L_D, such that transformations become less efficient the larger j is in the donor. Such a model would be expected to apply were transformation limited by cleavage of the imported DNA strand, or the scale or flexibility of the RecA-bound nucleoprotein filament involved in the recombination.

2. Deletion-limited transformation: $L = L_R$, $\lambda_I > 0$

Under this model, L corresponds to L_R, such that transformations become less efficient the larger j is in the recipient. Such a model would be expected to apply if transformation were limited by the scale or flexibility of the chromosomal locus involved in the recombination, or by the increasingly long time taken for the second homologous arm to locate a more distant binding site and anneal, after the first arm has bound.

3. Annealing-limited transformation: $L = 0$ or $\lambda_I = 0$

Under this model, neither L_D nor L_R contribute to L (or $\lambda_I = 0$), such that regions of heterology do not affect the efficiency of homologous recombination. Such a model would be expected to apply if transformation were limited by the annealing of homologous sequence, or the stability of the resulting heteroduplex complex, but unaffected by any intervening heterologous sequence.

4. Heterology-limited transformation: $L \alpha (L_D + L_R)$, $\lambda_I > 0$

Under this model, both L_D and L_R contribute to L, such that transformations become less efficient according to the size of j in both the donor and recipient. Such a model would be expected to apply if there were limitations imposed by the scale and flexibility of both the chromosomal locus and the RecA-coated nucleoprotein filament, or inhibition of exchange by the slower annealing of the second homologous arm as the separation of the flanking regions of similarity increases in terms of both the donor and recipient DNA.

Quantifying the rate of exchange

Using an analogous approach, the rate at which a single nucleotide polymorphism (SNP) S were acquired by transformation could be modelled as:

$$p(e\{S\}) = \sum_{d=h}^{C-h-1} 2\tau(1-\lambda_R)^{d+h}$$

Where h is the length of a homologous arm necessary to exchange a polymorphism within a region of sequence similarity. As the SNP, by definition, is in a homologous region, $L = 0$. Measuring this rate allows the effect of L on $e\{j\}$ to be estimated without directly measuring τ or H by estimating the ratio:

$$\frac{p(e\{j\})}{p(e\{S\})} = \frac{\sum_{d=H}^{C-H-1} 2\tau(1-\lambda_R)^{d+H}(1-\lambda_I)^L}{\sum_{d=h}^{C-h-1} 2\tau(1-\lambda_R)^{d+h}}$$

Assuming that H and h are independent of L (i.e. the same homologous arm lengths are involved in the exchange of any size of heterologous locus), a summary term τ_I can be introduced:

$$\tau_I = \frac{\sum_{d=H}^{C-H-1}(1-\lambda_R)^{d+H}}{\sum_{d=h}^{C-h-1}(1-\lambda_R)^{d+h}}$$

Allowing τ to vary with L, such that the relative transformation rate of each genotype g varies according to the parameter τ_g, results in a model of the relative rate at which regions of heterology and SNPs are acquired by transformation with the form:

$$\frac{p(e\{j\}|g)}{p(e\{S\}|g)} = \frac{\tau_g \tau_I (1-\lambda_I)^L}{\tau_g}$$

Where L can be defined in terms of L_R and L_D according to the four different models.

The model was fitted to the experimental data using measurements of both $e\{S\}$ and $e\{j\}$. The observed $e\{S\}$ for a particular genotype was assumed to reflect the transformation rate with which it was associated, τ_g:

$$p(e\{S\}) = \tau_g$$

This genotype-specific estimate was then used to inform estimation of the τ_I and λ_I parameters using the measured rates of $e\{j\}$, which were assumed to be independent of L:

$$p(e\{j\}) = \tau_g \tau_I (1-\lambda_I)^L$$

The observed number of transformants per millilitre that had exchanged sequence at j ($x_{j,L}$) or had acquired a SNP ($x_{S,L}$) for a given genotype were assumed to be Poisson distributed around the mean defined by the functions

above. For experiments using 1000 ng μL^{-1} of donor DNA, rather than 100 ng μL^{-1}, $x_{j,L}$ and $x_{S,L}$ were reduced ten-fold, a correction that assumed the quantity of added genomic DNA to be non-saturating. The estimates of τ_g for L = 20 kb shown in Fig S11, which include adjusted estimates of transformability from both donor DNA concentrations, indicate this assumption is justified.

Therefore the maximum log likelihood estimate, l, of τ_g, τ_I and λ_I given the data for n lengths of j from L_1 to L_n, with m biological replicates (q_1...q_m) of each, was achieved using the Brent method and the initial parameter estimates τ_I = 1, and λ_I = 0 bp^{-1} (τ_g was initialised as the mean of $x_{S,L}$ over the q_m replicates for genotype g), and the function:

$$\ell(\lambda_I, \tau_g, \tau_I; x_{j,L}, x_{S,L}) = \sum_{L=L_1}^{L_n}\left[\log\left(\tau_g\tau_I(1-\lambda_I)^L\right)\sum_{q=q_1}^{q_m} x_{j,L,q} - m\left(\tau_g\tau_I(1-\lambda_I)^L\right) + \log(\tau_g)\sum_{q=q_1}^{q_m} x_{S,L,q} - m(\tau_g)\right]$$

Bootstrapping was used to quantify the uncertainty in these estimates. The 93 paired measurements of $e_I\{j\}$ and $e\{S\}$ for the insertion of regions of heterology were resampled with replacement 100 times, and the model refitted to each of these datasets to identify the full range of estimates for τ_I, λ_I and τ_g. This process was repeated independently for the 90 paired measurements of $e_D\{j\}$ and $e\{S\}$ for the deletion of regions of heterology.

Quantifying the asymmetry of exchange

The asymmetry of transformation for length L, φ_L, can be defined as:

$$\varphi_L = \frac{e_{I,L}\{j\}e_{D,L}\{S\}}{e_{I,L}\{S\}e_{D,L}\{j\}}$$

Based on the data shown in Fig 2A, model 1 was assumed to apply to insertions. The relationship between L and $e_D\{j\}$ was less clear, and hence the change in $e_I\{j\}$

with L was assumed to dominate the magnitude of φ_L. Therefore the fitted function was of the form:

$$\varphi_L = \varphi_0(1 - \lambda_\varphi)^L$$

Where φ_0 is the theoretical asymmetry of a zero-length locus j, and λ_φ defines the change in asymmetry with L. Using the pairs of insertion and deletion experiments for each locus j of length L, the observed data were $x_{I,j,L}$ for insertion of locus j; $x_{I,S,L}$ for SNP transfer in the insertion experiment; $x_{D,j,L}$ for deletion of locus j; and $x_{D,S,L}$ for SNP transfer in the deletion experiment. For each L ($L_1 \ldots L_n$), there were m values ($q_1 \ldots q_m$), corresponding to all combinations of measurements of $x_{I,j,L}/x_{I,S,L}$ and $x_{D,j,L}/x_{D,S,L}$. Therefore the maximum log likelihood estimate, l, of φ_0 and λ_φ given the data for n lengths of j from L_1 to L_n was achieved through maximising the log likelihood, assuming measurements to be Poisson distributed around a mean value determined by the above formula, using the Brent method and initial parameter estimates $\varphi_0 = 1$ and $\lambda_\varphi = 10^{-9}$ bp^{-1}, and the function:

$$\ell\left(\lambda_\varphi, \varphi_0; x_{I,j,L}, x_{I,S,L}, x_{D,j,L}, x_{D,S,L}\right) = \sum_{L=L_1}^{L_n}\left[\log\left(\varphi_0(1-\lambda_\varphi)^L\right)\sum_{q=q_1}^{q_m}\frac{x_{I,j,L,q}\,x_{D,S,L,q}}{x_{I,S,L,q}\,x_{D,j,L,q}} - m\left(\varphi_0(1-\lambda_\varphi)^L\right)\right]$$

One hundred bootstrap replicates were again used to measure the variation in parameter estimates.

Text S2: Models for deletion of heterologous DNA by short fragments

With modification, the models described in Text S1 can be applied to the deletion of genomic islands by short DNA fragments. In this case, L can be assumed to be zero, as $e\{j\}$ appears to be insertion-limited and L_D = 0, based on the results in Fig 2. C represents the size of the PCR fragment, as this donor DNA is no longer circular; hence the furthest a recombination can start or end relative to j is $C/2$. In each of these models, the factor of two that results from recombinations being able to affect either strand of the genome is omitted, because the two halves of the DNA fragment are considered indistinguishable, and all experimental quantifications of rates are relative. Under these circumstances, four alternative models can be defined based on the nature of two key assumptions. The first assumption is that H is either a distance that must exceed a threshold on both sides of j to resolve a recombination (based on Fig 1A, $H = H_{5'} = H_{3'}$), or $2H$ is a total distance that may be distributed across j in an unbalanced manner ($2H = H_{5'} + H_{3'}$). The second assumption regards the termination of recombination. One hypothesis is that a process must actively terminate the recombination before the end of the added donor DNA fragment. This may represent nicking of the DNA on its import, or strand processing at a crossover. Alternatively, no active termination may need to be instigated, and instead successful resolution may automatically be triggered upon reaching the end of the added donor DNA fragment.

For the experiments shown in Fig 3A, j is centrally located within C. For a recombination initiating d bases from j at i, which is constrained to be between one and $C/2$ bases from j, the probability that the recombination reaches j is:

$$p(r\{d\}) = (1 - \lambda_R)^d$$

For the exchange of j to occur, corresponding to a deletion and the restoration of an intact *tetM* gene in this case, the recombination must also span $H_{3'}$ downstream of j. This is assumed to correspond to:

$$p(r\{H_{3'}\}) = (1 - \lambda_R)^H$$

Assuming the homologous arms are balanced across j, as in the model described in Text S1, both $H_{5'}$ and $H_{3'}$ must be at least H in length. Therefore:

$$H_{3'} \geq H$$

However, if the homologous arms can be unbalanced across j, then $H_{5'}$ and $H_{3'}$ must instead sum to at least $2H$. As d corresponds to $H_{5'}$ (Fig 1), this can be expressed as:

$$H_{3'} \geq 2H - d$$

These two probabilities are important in defining $p(e_D\{j\})$ in these experiments. However, as H is no longer negligible relative to C, the implicit assumption that any recombination modelled as extending beyond C is automatically curtailed at this maximum length is potentially problematic. It is alternatively possible that recombinations can lead to the exchange of j only if they successfully terminate within the boundaries of the donor DNA. The probability of a recombination terminating prior to reaching the end of the fragment requires the length of the recombination on the 3' side of j, $l_{3'}$, to be both greater than $H_{3'}$ and less than, or equal to, $C/2$:

$$p\left(H_{3'} \leq l_{3'} \leq \frac{C}{2}\right) = (1 - \lambda_R)^{H_{3'}} - (1 - \lambda_R)^{\frac{C}{2}}$$

These component probabilities can then be combined to give four different models, two of which assume H is balanced across j, and two of which assume the recombination must terminate within the boundaries of the donor fragment of length C.

1. **Balanced *H*, unterminated recombination**

Under this model, a recombination that deletes the region of heterology at locus j, $e_D\{j\}$, must initiate at least H bases from j, and be able to span H bases on the other side. Therefore $e_D\{j\}$ requires $H_{3'} \geq H$ and $l_{3'} \geq H_{3'}$:

$$p(e_D\{j\})\ \alpha\ (1-\lambda_R)^d(1-\lambda_R)^H \text{ for } H \leq d \leq C/2$$

Under the condition $C \geq 2H$, this implies:

$$p(e_D\{j\}|C \geq 2H)\ \alpha\ \sum_{d=H}^{C/2}(1-\lambda_R)^{d+H}$$

The plotted values in Fig 3A correspond to a ratio of the frequency of successful recombinations with a fragment of length f (corresponding to C in the above relationships) relative to that same frequency with the fragment of the maximal length, M, which was 2 kb in this experiment. The factor f/M was used to correct for the number of DNA molecules available for transformation, because all fragments were added at a concentration of 60 ng μL^{-1}, thereby assuming that the competence system was not saturated by the amount of DNA substrate in the transformation reaction. This appears accurate, based on the data for transformations using 100 ng μL^{-1} and 1000 ng μL^{-1} donor DNA, as there was a corresponding ten-fold increase in the number of observed transformants at the elevated DNA concentration. Therefore, in order to fit this model to experimental data, the ratio for the balanced H unterminated recombination model, $y_{f,bu}$, can be expressed as:

$$y_{f,bu} = \frac{f\sum_{d=H}^{f/2}(1-\lambda_R)^{d+H}}{M\sum_{d=H}^{M/2}(1-\lambda_R)^{d+H}}$$

2. **Balanced *H*, terminated recombination**

Under this model, $e_D\{j\}$ requires a recombination initiating at least H bases from j, and both span H bases, and terminate prior to reaching $C/2$ bases, from j on the other side. Therefore $e_D\{j\}$ requires $H_{3'} \geq H$ and $C/2 \geq l_{3'} \geq H_{3'}$:

$$p(e_D\{j\}) \propto (1-\lambda_R)^d\left[(1-\lambda_R)^H - (1-\lambda_R)^{C/2}\right]$$
$$\text{for } H \leq d \leq C/2$$

Which implies:

$$p(e_D\{j\}|\mathrm{C} \geq 2\mathrm{H}) \propto \sum_{d=H}^{C/2}\left[(1-\lambda_R)^{d+H} - (1-\lambda_R)^{d+\frac{C}{2}}\right]$$

With the DNA molecule ratio correction, this ratio for the balanced *H* terminated recombination model, $y_{f,bt}$, for a fragment of length *f* relative to one of length *M* can therefore be expressed as:

$$y_{f,bt} = \frac{f\sum_{d=H}^{f/2}\left[(1-\lambda_R)^{d+H} - (1-\lambda_R)^{d+\frac{f}{2}}\right]}{M\sum_{d=H}^{M/2}\left[(1-\lambda_R)^{d+H} - (1-\lambda_R)^{d+\frac{M}{2}}\right]}$$

3. Unbalanced *H*, unterminated recombination

Under this model, $e_D\{j\}$ requires a recombination that initiates upstream of *j* and at least 2*H* bases from the distal end of the DNA fragment. Hence *i* may be either directly adjacent to *j* if $C/2 > 2H$, or at least $2H$-$C/2$ bases from *j*, if $C/2 < 2H$. Therefore $e_D\{j\}$ requires $H_{3'} \geq 2H-d$ and $l_{3'} \geq H_{3'}$. Where *d* was greater than 2*H*, then the recombination need only span from *i* to *j*:

$$p(e_D\{j\}) \propto (1-\lambda_R)^d \text{ for } 2H \leq d \leq C/2$$

However, if $d < 2H$, then the recombination also needed to span $H_{3'}$ bases on the distal side of *j*, calculated as $2H$-d:

$$p(e_D\{j\}|\mathrm{C} \geq 2\mathrm{H}) \propto (1-\lambda_R)^d(1-\lambda_R)^{2H-d} \text{ for } \max(1,2H\text{-}C/2) \leq d < 2H$$

This implies the combined probability of spanning d on the 5' side of j and $2H$-d on the 3' side of j is:

$$p(e_D\{j\}|\mathrm{C} \geq 2\mathrm{H})\ \alpha\ (1-\lambda_R)^{2H} \text{ for } \max(1,2H\text{-}C/2) \leq d < 2H$$

Which implies an overall probability of:

$$p(e\{j\}|\mathrm{C} \geq 2\mathrm{H})\ \alpha \sum_{d=\max\left(1,2H-\frac{C}{2}\right)}^{2H-1} (1-\lambda_R)^{2H} + \sum_{d=2H}^{C/2} (1-\lambda_R)^{d}$$

With the DNA molecule ratio correction, the ratio of deletions with a fragment of length f relative to those with a fragment of length M with the unbalanced H unterminated recombination model, $y_{f,uu}$, can therefore be expressed as:

$$y_{f,uu} = \frac{f\left[\sum_{d=\max\left(1,2H-\frac{f}{2}\right)}^{2H-1} (1-\lambda_R)^{2H} + \sum_{d=2H}^{\frac{f}{2}} (1-\lambda_R)^{d}\right]}{M\left[\sum_{d=\max\left(1,2H-\frac{M}{2}\right)}^{2H-1} (1-\lambda_R)^{2H} + \sum_{d=2H}^{\frac{M}{2}} (1-\lambda_R)^{d}\right]}$$

4. **Unbalanced *H*, terminated recombination**

Under this model, $e_D\{j\}$ requires a recombination that initiates at least $2H$ bases from the distal end of the DNA fragment, which may be either directly adjacent to j if $C/2 > 2H$, or at least $2H$-$C/2$ bases from j, if $C/2 < 2H$. The recombination must also terminate prior to reaching the distal end of the DNA fragment. Therefore $e_D\{j\}$ requires $H_{3'} \geq 2H$-d and $C/2 \geq l_{3'} \geq H_{3'}$. Where d was greater than $2H$, then the recombination need only span from i to j, and terminate between j and the distal end of f, $C/2$ bases away:

$$p(e_D\{j\}|C \geq 2H)\ \alpha\ (1-\lambda_R)^{d}\left[1-(1-\lambda_R)^{\frac{C}{2}}\right] \text{ for } 2H \leq d \leq C/2$$

Which simplifies to:

$$p(e_D\{j\}|C \geq 2H)\ \alpha\ (1-\lambda_R)^d - (1-\lambda_R)^{d+\frac{C}{2}} \text{ for } 2H \leq d \leq C/2$$

However, if $d < 2H$, then the recombination also needed to span $H_{3'}$ bases on the distal side of j, calculated as $2H$-d, and terminate prior to reaching the distal end of the DNA fragment:

$$p(e_D\{j\}|C \geq 2H)\ \alpha\ (1-\lambda_R)^d \left[(1-\lambda_R)^{2H-d} - (1-\lambda_R)^{\frac{C}{2}}\right]$$
$$\text{for } \max(1{,}2H\text{-}C/2) \leq d \leq 2H$$

Which simplifies to:

$$p(e_D\{j\}|C \geq 2H)\ \alpha\ (1-\lambda_R)^{2H} - (1-\lambda_R)^{d+\frac{C}{2}} \text{ for } \max(1{,}2H\text{-}C/2) \leq d \leq 2H$$

Which implies:

$$p(e_D\{j\}|\ C \geq 2H)\ \alpha$$
$$\sum_{d=\max(1,2H-\frac{C}{2})}^{2H-1}\left[(1-\lambda_R)^{2H} - (1-\lambda_R)^{d+\frac{C}{2}}\right] + \sum_{d=2H}^{\frac{C}{2}}\left[(1-\lambda_R)^{d} - (1-\lambda_R)^{d+\frac{C}{2}}\right]$$

With the DNA molecule ratio correction, this ratio for the unbalanced H terminated recombination model, $y_{f,ut}$, for a fragment of length f relative to one of length M can therefore be expressed as:

$$y_{f,ut} = \frac{f}{M}\left[\frac{\sum_{d=\max(1,2H-\frac{f}{2})}^{2H-1}\left[(1-\lambda_R)^{2H} - (1-\lambda_R)^{d+\frac{f}{2}}\right] + \sum_{d=2H}^{f/2}\left[(1-\lambda_R)^{d} - (1-\lambda_R)^{d+\frac{f}{2}}\right]}{\sum_{d=\max(1,2H-\frac{M}{2})}^{2H-1}\left[(1-\lambda_R)^{2H} - (1-\lambda_R)^{d+\frac{M}{2}}\right] + \sum_{d=2H}^{M/2}\left[(1-\lambda_R)^{d} - (1-\lambda_R)^{d+\frac{M}{2}}\right]}\right]$$

Parameter estimation

Data consisted of $x_{j,f}$, the frequency with which *j* is deleted with a fragment of length *f*; $x_{S,f}$, the rate of SNP acquisition in the same experiment; $\bar{x}_{j,M}$, the mean frequency with which *j* is deleted with a fragment of length *M*, and $\bar{x}_{S,M}$, the mean rate of SNP acquisition in the same experiment. With the DNA molecule ratio correction, these were processed to generate a metric y_f:

$$y_f = \frac{f x_{j,f} \bar{x}_{S,M}}{M x_{S,f} \bar{x}_{j,M}}$$

The four models were fitted to data through minimising the square difference between the log transformed predicted and observed results. Model fitting used the 'maxSANN' function of the maxLik R package (Henningsen and Toomet 2011). Fits were conducted on the overall dataset (Fig 3B) and the individual datasets (Fig S14) using the initial values of λ_I = 0.0005 bp^{-1} and *H* = 250 bp (Table S1). Although y_f values of zero were kept for model fitting, this was substituted for an arbitrary value of 7.5x10^{-6} for plotting both experimental data and simulation outputs (Fig 3 & S14).

Text S3: Models for deletion of heterologous DNA by unbalanced short fragments

The models described in Text S2 for the deletion of genomic islands by short DNA fragments that span a locus *j* in a balanced manner can be modified to apply to short DNA fragments that span *j* in an unbalanced manner. The length of the fragment, *C*, can be split into a segment of unchanging length *u* on one side of *j*, and a segment of variable length *v* on the other side. The same four models as in Text S2 can therefore be redefined in the following manner.

1. Balanced *H*, unterminated recombination

Based on the relationship defined in Text S2:

$$p(e_D\{j\})\ \alpha\ (1-\lambda_R)^d(1-\lambda_R)^H \text{ for } H \le d \le u \text{ and } H \le d \le v$$

For a fragment in which both *u* and *v* are of length at least *H*, this implies:

$$p(e_D\{j\}|\ u \ge H \cap v \ge H)\ \alpha \sum_{d=H}^{u}(1-\lambda_R)^{d+H} + \sum_{d=H}^{v}(1-\lambda_R)^{d+H}$$

Denoting the maximum length of *v* as *M*, and adding a DNA molecule ratio correction, the plotted ratio y_f for this balanced, unterminated model ($y_{f,bu}$, where $f = u + v$) is therefore:

$$y_{f,bu} = \frac{(u+v)}{(u+M)}\left[\frac{\sum_{d=H}^{u}(1-\lambda_R)^{d+H} + \sum_{d=H}^{v}(1-\lambda_R)^{d+H}}{\sum_{d=H}^{u}(1-\lambda_R)^{d+H} + \sum_{d=H}^{M}(1-\lambda_R)^{d+H}}\right]$$

When plotted, this has an unusual form with an abrupt change in $y_{f,bu}$, where $v = H$ if $u > v$, as recombinations transition from being impossible to relatively efficient at this boundary.

2. Balanced *H*, terminated recombination

Based on the relationship defined in Text S2:

$$p(e_D\{j\})\ \alpha\ (1-\lambda_R)^d[(1-\lambda_R)^H-(1-\lambda_R)^v]$$
for $H \leq d \leq u$

$$p(e_D\{j\})\ \alpha\ (1-\lambda_R)^d[(1-\lambda_R)^H-(1-\lambda_R)^u]$$
for $H \leq d \leq v$

Which implies:

$$p(e_D\{j\}|\ u \geq H \cap v \geq H)\ \alpha\ \sum_{d=H}^{u}[(1-\lambda_R)^{d+H}-(1-\lambda_R)^{d+v}] + \sum_{d=H}^{v}[(1-\lambda_R)^{d+H}-(1-\lambda_R)^{d+u}]$$

With the DNA molecule ratio correction, the ratio $y_{f,bt}$ can therefore be expressed as:

$$y_{f,bt} = \frac{(u+v)}{(u+M)}\left[\frac{\sum_{d=H}^{u}[(1-\lambda_R)^{d+H}-(1-\lambda_R)^{d+v}]+\sum_{d=H}^{v}[(1-\lambda_R)^{d+H}-(1-\lambda_R)^{d+u}]}{\sum_{d=H}^{u}[(1-\lambda_R)^{d+H}-(1-\lambda_R)^{d+M}]+\sum_{d=H}^{M}[(1-\lambda_R)^{d+H}-(1-\lambda_R)^{d+u}]}\right]$$

3. **Unbalanced *H*, unterminated recombination**

Based on the relationship defined in Text S2:

$$p(e_D\{j\})\ \alpha\ (1-\lambda_R)^d \text{ for } 2H \leq d \leq u \text{ and } 2H \leq d \leq v$$

And:

$$p(e_D\{j\})\ \alpha\ (1-\lambda_R)^d(1-\lambda_R)^{2H-d}$$

for max(1,2*H*-*u*) ≤ *d* ≤ 2*H* and max(1,2*H*-*v*) ≤ *d* ≤ 2*H*

Which simplifies to:

$$p(e_D\{j\})\ \alpha\ (1-\lambda_R)^{2H} \text{ for } \max(1,2H\text{-}u) \le d \le 2H \text{ and } \max(1,2H\text{-}v) \le d \le 2H$$

Which implies:

$$p(e_D\{j\}|\ (u+v) \ge 2H)\ \alpha \sum_{d=\max(1,2H-v)}^{d=2H-1}(1-\lambda_R)^{2H} + \sum_{d=2H}^{d=u}(1-\lambda_R)^{d} + \sum_{d=\max\,(1,2H-u)}^{d=2H-1}(1-\lambda_R)^{2H} + \sum_{d=2H}^{d=v}(1-\lambda_R)^{d}$$

With the DNA molecule ratio correction, the ratio $y_{f,uu}$, can therefore be expressed as:

$$y_{f,uu} = \frac{(u+v)}{(u+M)}\left[\frac{\sum_{d=\max\,(1,2H-v)}^{2H-1}(1-\lambda_R)^{2H} + \sum_{d=2H}^{u}(1-\lambda_R)^{d} + \sum_{d=\max\,(1,2H-u)}^{2H-1}(1-\lambda_R)^{2H} + \sum_{d=2H}^{v}(1-\lambda_R)^{d}}{\sum_{d=\max\,(1,2H-M)}^{2H-1}(1-\lambda_R)^{2H} + \sum_{d=2H}^{u}(1-\lambda_R)^{d} + \sum_{d=\max\,(1,2H-u)}^{2H-1}(1-\lambda_R)^{2H} + \sum_{d=2H}^{M}(1-\lambda_R)^{d}}\right]$$

4. Unbalanced *H*, terminated recombination

Based on the relationship defined in Text S3:

$$p(e_D\{j\})\ \alpha\ (1-\lambda_R)^d[1-(1-\lambda_R)^v] \text{ for } 2H \le d \le u$$

$$p(e_D\{j\})\ \alpha\ (1-\lambda_R)^d[1-(1-\lambda_R)^u] \text{ for } 2H \le d \le v$$

And:

$$p(e_D\{j\})\ \alpha\ (1-\lambda_R)^d[(1-\lambda_R)^{2H-d}-(1-\lambda_R)^v]$$
$$\text{for } \max(1,2H\text{-}v) \le d < 2H$$

$$p(e_D\{j\})\ \alpha\ (1-\lambda_R)^d[(1-\lambda_R)^{2H-d}-(1-\lambda_R)^u]$$

for $\max(1,2H\text{-}u) \leq d < 2H$

Which implies:

$$p(e_D\{j\}|\ (u+v) \geq 2H)\ \alpha$$

$$\sum_{d=\max(1,2H-v)}^{2H-1} [(1-\lambda_R)^{2H} - (1-\lambda_R)^{d+v}] + \sum_{d=2H}^{u} [(1-\lambda_R)^{d} - (1-\lambda_R)^{d+v}]$$
$$+ \sum_{d=\max(1,2H-u)}^{2H-1} [(1-\lambda_R)^{2H} - (1-\lambda_R)^{d+u}]$$
$$+ \sum_{d=2H}^{v} [(1-\lambda_R)^{d} - (1-\lambda_R)^{d+u}]$$

With the DNA molecule ratio correction, the ratio $y_{f,ut}$ can therefore be expressed as:

$$y_{f,ut} = \frac{(u+v)}{(u+M)} \left[\frac{(u+v)\sum_{d=\max(1,2H-v)}^{d=2H-1}[(1-\lambda_R)^{2H} - (1-\lambda_R)^{d+v}] + \sum_{d=2H}^{d=u}[(1-\lambda_R)^{d} - (1-\lambda_R)^{d+v}] + \sum_{d=\max(1,2H-u)}^{d=2H-1}[(1-\lambda_R)^{2H-d} - (1-\lambda_R)^{d+u}] + \sum_{d=2H}^{d=v}(1-\lambda_R) \text{[illegible]}}{(u+M)\sum_{d=\max(1,2H-M)}^{d=2H-1}[(1-\lambda_R)^{2H} - (1-\lambda_R)^{d+M}] + \sum_{d=2H}^{d=M}[(1-\lambda_R)^{d} - (1-\lambda_R)^{d+M}] + \sum_{d=\max(1,2H-u)}^{d=2H-1}[(1-\lambda_R)^{2H-d} - (1-\lambda_R)^{d+u}] + \sum_{d=2H}^{d=M}(1-\lambda_R \text{[illegible]}} \right.$$

Parameter estimation

Parameters were estimated as described in Text S2.

Text S4: Genetic constructs used in the transformation assays

Construction of *S. pneumoniae* R6I

S. pneumoniae R6x (Tiraby and Fox 1973) was first transformed with an *rpsL** allele containing a spontaneous streptomycin resistance mutation, followed by selection on 100 µg mL^{-1} streptomycin sulphate (Sigma-Aldrich). To simultaneously remove the phase variable *ivr* restriction modification locus (Croucher et al. 2014) and introduce ICE*Sp*23FST81 (Croucher et al. 2009), 1 kb sequences flanking the R6x *ivr* locus were amplified by PCR using RedTaq (Sigma-Aldrich), according to manufacturer's instructions, and primers ivr.LL, ivr.CRA, ivr.CL and ivr.RR (Invitrogen; Table S2) at a final concentration of 10 µM. The Janus cassette (Sung et al. 2001) was amplified using Jns.F and Jns.R (Invitrogen) and purified through agarose gel electrophoresis and the GenElute Gel Extraction Kit (Sigma-Aldrich). Five hundred nanograms of each amplicon was digested with *Apa*I (Promega) (ivr.LL-ivr.CRA), *Bam*HI (Promega) (ivr.CL-ivr.RR), or both (Janus cassette) for two hours at 37°C, followed by 15 min at 65°C to deactivate the enzymes. Five microlitres of each of the three DNA fragments were ligated using T4 DNA ligase (Invitrogen) at 25°C for two hours, followed by 15 minutes of ligase deactivation at 74°C. The full construct was amplified and purified, then used to transform *S. pneumoniae* R6x *rpsL**. Candidate *ivr*::Janus mutants were selected on 400 µg mL^{-1} kanamycin sulphate (Sigma-Aldrich), and verified through PCR and testing streptomycin sensitivity.

Transconjugation of ICE*Sp*23FST81 required filter mating through concentrating 1 mL stationary phase culture samples of ATCC 700669 and R6x *rpsL* ivr::*Janus onto a 0.45 µm pore MF membrane filter (Sigma-Aldrich). The filter was incubated for 24 h at 35°C on a non-selective THY agar plate containing 5% defibrinated horse blood (E&O Laboratories) while covered with CAT-THY agar (Shoemaker et al. 1980) containing 50 µL DNase I (New England Biolabs). Candidate transconjugants were selected from the cell mass through 48 h incubation on THY agar containing 400 µg mL^{-1} kanamycin sulphate and 10 µg mL^{-1} tetracycline hydrochloride (Sigma-Aldrich). Sensitivity to streptomycin, colony morphology and PCR genotyping were used to verify candidates

represented the insertion of ICE*Sp*23FST81 at the att_{rplL} site in R6x *rpsL** *ivr::*Janus.

To remove the Janus cassette, the same regions flanking the *ivr* locus were amplified as previously, except primer ivr.CRB was used instead of ivr.CRA. The two DNA fragments were digested using BamHI, ligated, and the full construct amplified by PCR and purified. This construct was used to transform *S. pneumoniae* R6x *rpsL** *ivr::*Janus att_{rplL}::[ICE*Sp*23FST81] and candidate colonies selected using 100 μg mL^{-1} streptomycin sulphate. Removal of Janus was verified by PCR and testing kanamycin sensitivity. This final *S. pneumoniae* R6x *rpsL* Δivr* att_{rplL}::[ICE*Sp*23FST81] genotype contained a substantial insertion, hence was renamed R6I.

Construction of R6I derivatives

The *ermB* gene was amplified from *S. pneumoniae* 11930 (Croucher et al. 2011) using primers ermB.LA and ermB.RB (Table S2) and ligated between two halves of the *tetM* gene from ICE*Sp*23FST81, amplified using primers tet.LL, tet.CRA and tet.CLB, tetRR. The construct was then used to transform R6I to produce strain R6I *tetM::ermB* (R6I-1*i*). A second strain was derived from R6I *tetM::ermB* through transformation with a construct consisting of the 5' end of *tetM* (primers: tet.LL, tet.CRA), the *aph3'* gene amplified from the genome of *S. pneumoniae* TIGR4Δ*cps* (Pearce et al. 2002) (primers: kan.L, kan.R), and the *ermB* gene (primers: ermB.LB, ermB.R). The consequent R6I *tetM*::[*aph3', ermB*] strain (R6I-2*i*) was isolated following selection with kanamycin sulphate. *S. pneumoniae* R6I itself therefore served as R6I-1*d* and R6I-2*d*.

A modified *ermB* construct was used for the preparation of genotypes for *L* greater than 2 kb, with one different homologous arm (generated with primers 2A and 2B) used to remove of the 5' half of *tetM*, generating the tetracycline-sensitive R6I Δ$tetM_{5'}$ *ermB*. The 5' half of *tetM* was amplified from R6I with primers tet.LLX and tet.CRA, then ligated to the *aph3'* gene, amplified from the genome of *S. pneumoniae* TIGR4Δ*cps* (Pearce et al. 2002) with primers kan.L and kan.R. The resultant construct ('TK') was used to prepare six constructs that

were used to introduce the 5' half of *tetM* at different positions upstream of its original location. In each case, 2 kb genomic regions located at increasing distances from the *3'* half of the *tetM* gene (tet.CLB binding site) were amplified as 1 kb fragments, which were ligated to either end of the TK construct using *Xba*I and *Bam*HI restriction sites added using the relevant primers (Table S2). The resulting six TK-based constructs were used to transform R6I $\Delta tetM_{5'}$ *ermB* to generate R6I *tetM::*[*aph3', ermB, L*] strains, where *L* is the length of the sequence separating the two halves of the *tetM* gene (including the *ermB* and *aph3'* genes themselves), corresponding to the R6I-*Li* genotypes.

For each of these, the insertions flanked by *aph3'* and *ermB* were removed by transforming the R6I *tetM::*[*aph3', ermB, L*] strain with the *tetM* sequence amplified from R6I using primers tetM.L and tetM.R, followed by selection on 10 μg mL^{-1} tetracycline hydrochloride. These experiments generated the strains R6I *ΔL*, where *L* is the length of the insert removed, corresponding to the R6I-*Ld* genotypes. For each R6I *tetM::*[*aph3', ermB, L*] and R6I *ΔL* strain, rifampicin-resistant mutants were generated through transformation with a PCR amplicon of a $rpoB_{S482F}$ allele. This was generated with primers rpoB.L and rpoB.R from a spontaneous mutant in which a C→T base substitution caused a S482F amino acid substitution. Selection of transformants used THY agar plates supplemented with 4 μg mL^{-1} rifampicin (Alfa Aesar). Genomic DNA (gDNA) was extracted from these R6I *tetM*::[*aph3', ermB, L*] $rpoB_{S482F}$ and R6I Δ*L* $rpoB_{S482F}$ strains using the Wizard Genomic DNA Purification Kit (Promega).

Fragments of *tetM* used in transformation assays

To quantify the rate of deletion with *tetM* fragments of different lengths, R6I-*Li* genotypes were transformed with PCR amplicons using the approach described for the gDNA transformations. Each *tetM* fragment was amplified with the primers indicated in Fig S13 and Table S2 according to the experimental design, gel purified with the GenElute Gel Extraction kit (Sigma-Aldrich), and standardised at a concentration of 60 ng μL^{-1}. Similarly, gDNA from a rifampicin-resistant *S. pneumoniae* TIGR4Δ*cps* genotype (Pearce et al. 2002) was extracted and diluted to 60 ng μL^{-1}. Five microliters of a ten-fold dilution of the appropriate

tetM fragment, and the rifampicin-resistance encoding gDNA, were each added to the 1 mL transformation. Of this volume, 10 µL was spread on THY plates with 4 µg mL^{-1} rifampicin, to count colonies to calculate $e\{S\}$, and between 100 and 900 µL spread on THY plates supplemented with 10 µg mL^{-1} tetracycline hydrochloride, to count colonies to quantify $e_D\{j\}$.

1 **Supplementary Figures**

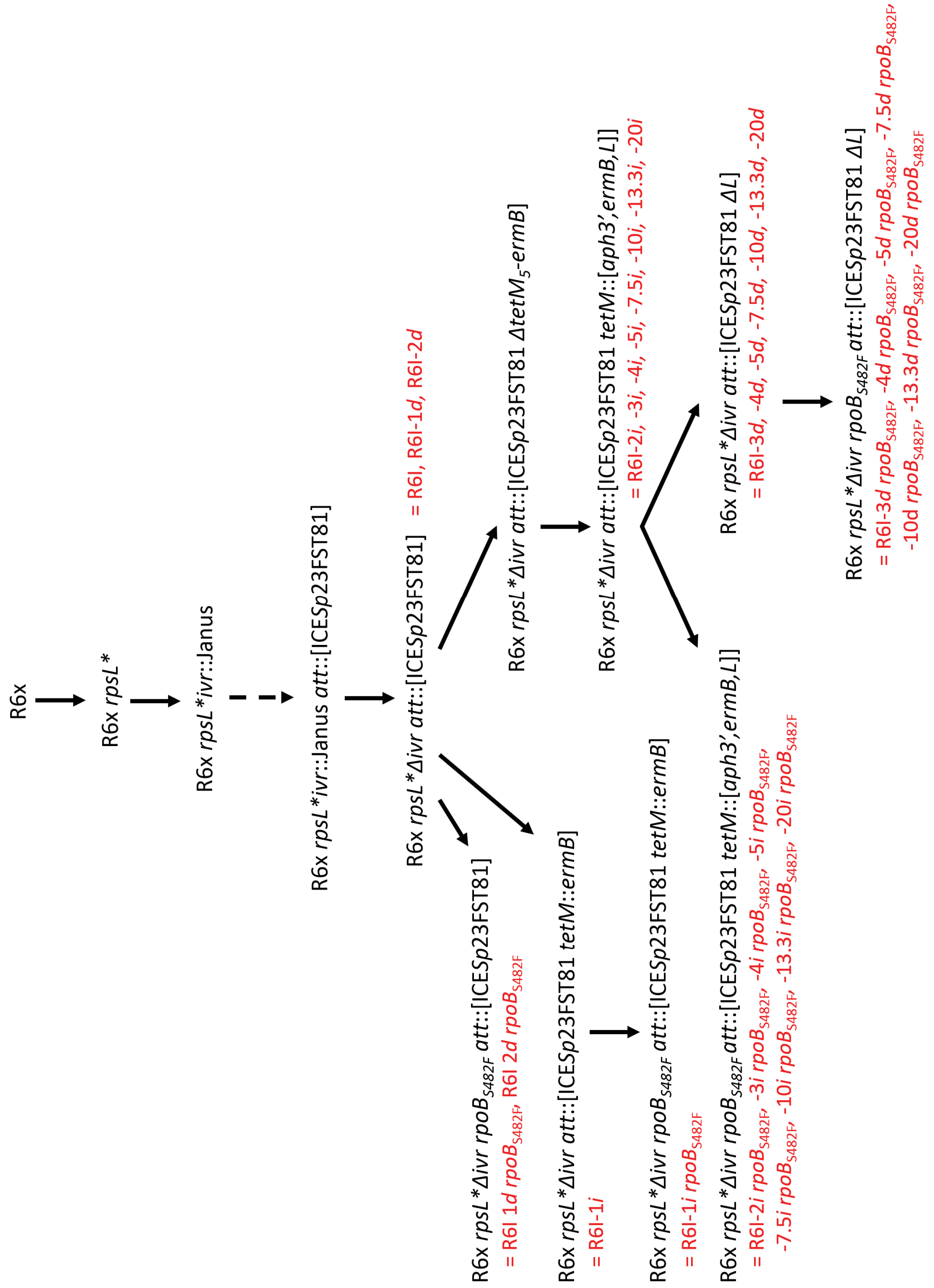

2

3 **Figure S1** – Construction of the genotypes used in this study. This diagram
4 shows the stages by which the genotypes used in the described experiments
5 were generated from the progenitor *S. pneumoniae* R6x strain. The names in red
6 are those used in the main text; those in black are the formal genotype
7 descriptions. Solid or dashed arrows indicate that the relevant strain was
8 produced via transformation or transconjugation, respectively.

(A)

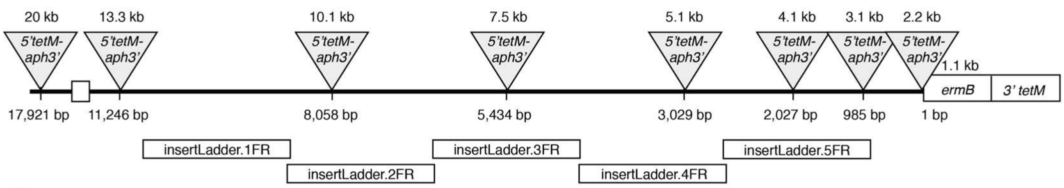

(B)

L (kb)	Expected size of PCR products (bp): insertion/deletion genotypes					*tetM*.LR PCR product size (bp)
1	1977 / 1977	2135 / 2135	2079 / 2079	2145 / 2145	1646 / 1646	3228 / 2176
2	1977 / 1977	2135 / 2135	2079 / 2079	2145 / 2145	1646 / 1646	4340 / 2176
3	1977 / 1977	2135 / 2135	2079 / 2079	2145 / 2145	3810 / -	5264 / 2176
4	1977 / 1977	2135 / 2135	2079 / 2079	2145 / 2145	3810 / -	6306 / 2176
5	1977 / 1977	2135 / 2135	2079 / 2079	4309 / -	1646 / -	7308 / 2176
7.5	1977 / 1977	2135 / 2135	4243 / -	2145 / -	1646 / -	9713 / 2176
10	1977 / 1977	4299 / -	2079 / -	2145 / -	1646 / -	12337 / 2176
13.3	1977 / -	2235 / -	2079 / -	2145 / -	1646 / -	15525 / 2176
20	1977 / -	2235 / -	2079 / -	2145 / -	1646 / -	22200 / 2176

Figure S2 – PCR scheme for genotype validation. (A) Diagram showing the extent of PCR amplicons. The solid bar indicates the region of ICE*Sp*23FST81 involved in the insertion and deletion experiments. The positioning of different 'insertLadder' primer pairs, listed as separate forward ('F') and reverse ('R') primers in Table S2, relative to the 5' end of *ermB* is indicated under the bar by white rectangles. The grey triangles and numbers indicate the position of the *tetM*$_{5'}$*-aph3'* insertions in the different R6I-*Li* strains, again relative to the 5' end of *ermB*. (B) Five of the columns in the table correspond to the five insertLadder primer pairs in the panel above. Each row corresponds to a particular *L*. Within each cell, the two numbers are the predicted PCR amplicon sizes for the corresponding pair of R6I-*L*i and R6I-*L*d strains. All PCRs used an elongation time of two minutes; therefore products greater than 2.2 kb were not always amplified and are marked in blue. Dashes indicate PCRs that were not expected to yield a band. The rightmost column shows predicted sizes of amplicons amplified using the tetM.LR primer pair, but does not directly correspond to the panel above.

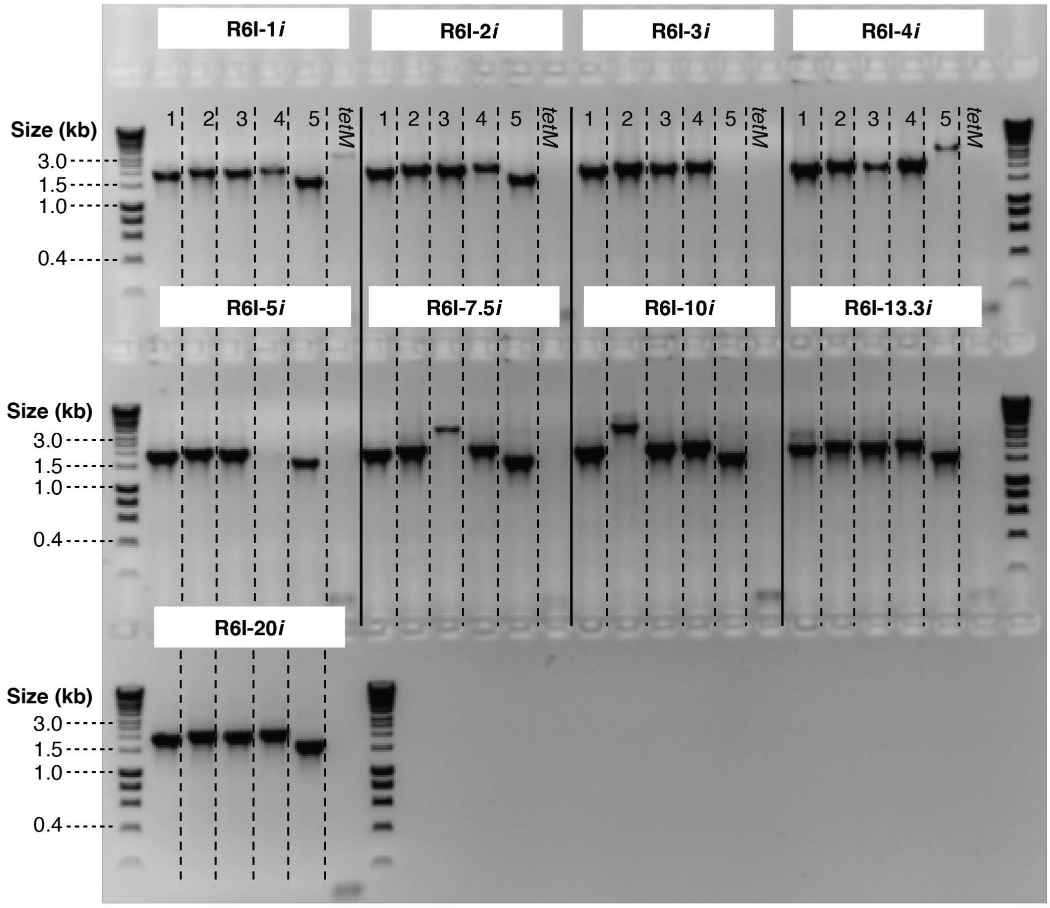

Figure S3 – Validating the construction of R6I-*Li* genotypes. As described in Fig S1, strains 2*i,* 3*i,* 4*i,* 5*i,* 7.5*i,* 10*i,* 13.3*i* and 20*i* were generated by transforming R6x *rpsL*Δivr* att_{rplL}::[ICE*Sp*23FST81] with *aph3'*- and *ermB*-containing constructs. Strain 1*i* was generated through the insertion of *ermB* only into the same background. The identity of the R6I-*Li* candidates was confirmed by running a set of validation PCRs on their genomic DNA (Fig S2), using primers insertLadder.1FR ('1'), insertLadder.2FR ('2'), insertLadder.3FR ('3'), insertLadder.4FR ('4'), insertLadder.5FR ('5'), and tetM.LR ('tetM'), the products of which were visualised on 1% agarose gel. Each lane contains 5 μL of PCR product or Hyperladder 1kb (Bioline), used as a DNA size marker. The PCR products correspond to the predicted sizes shown in Fig S2, confirming the presence of the expected lengths of DNA following transformation with the respective *aph3'* and *ermB* constructs.

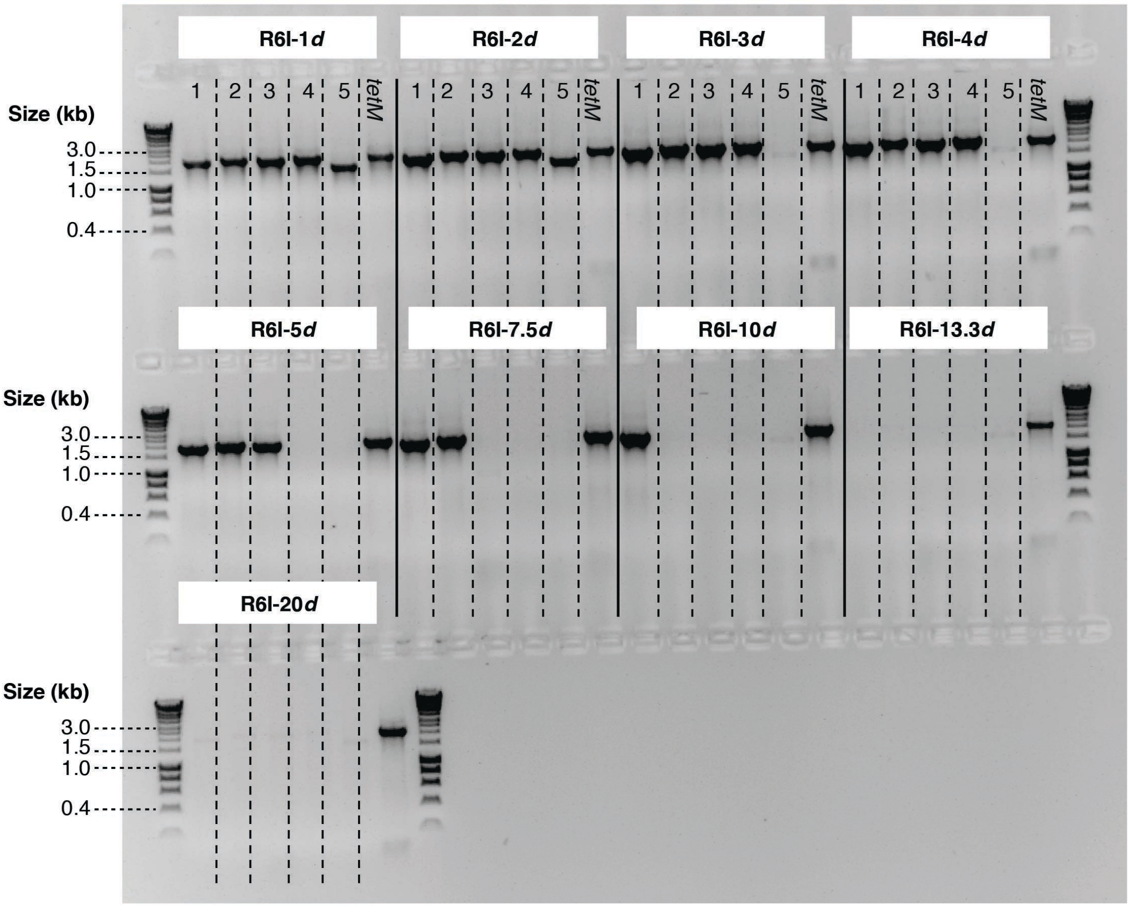

Figure S4 – Validating the construction of R6I-*Ld* genotypes. As described in Fig S1, strain R6I served as R6I-1*d* and R6I-2*d*. Strains R6I-3*d,* 4*d,* 5*d,* 7.5*d,* 10*d,* 13.3*d* and 20*d* were generated by transforming the corresponding R6x *rpsL*Δivr* *att*$_{rplL}$::[ICE*Sp*23FST81 *tetM*::[*aph3',ermB,L*]] genotype with the tetM.LR PCR product. These transformations aimed to remove *L* kb DNA inserted between the two halves of the *tetM* gene. The genotypes of these R6I-*Ld* candidates was verified by running a set of validation PCRs on their genomic DNA (Fig S2), using primer pairs insertLadder.1FR ('1'), insertLadder.2FR ('2'), insertLadder.3FR ('3'), insertLadder.4FR ('4'), insertLadder.5FR ('5'), and tetM.LR ('*tetM*'). The figure shows a 1% agarose gel in which each lane contains 5 μL of PCR product or DNA size marker Hyperladder 1kb (Bioline). As the PCR products correspond to the predicted sizes outlined in Fig S2, confirming that the *L* kb of DNA originally separating the two halves of *tetM* were removed successfully. These strains were used as recipients in experiments shown in Fig 2.

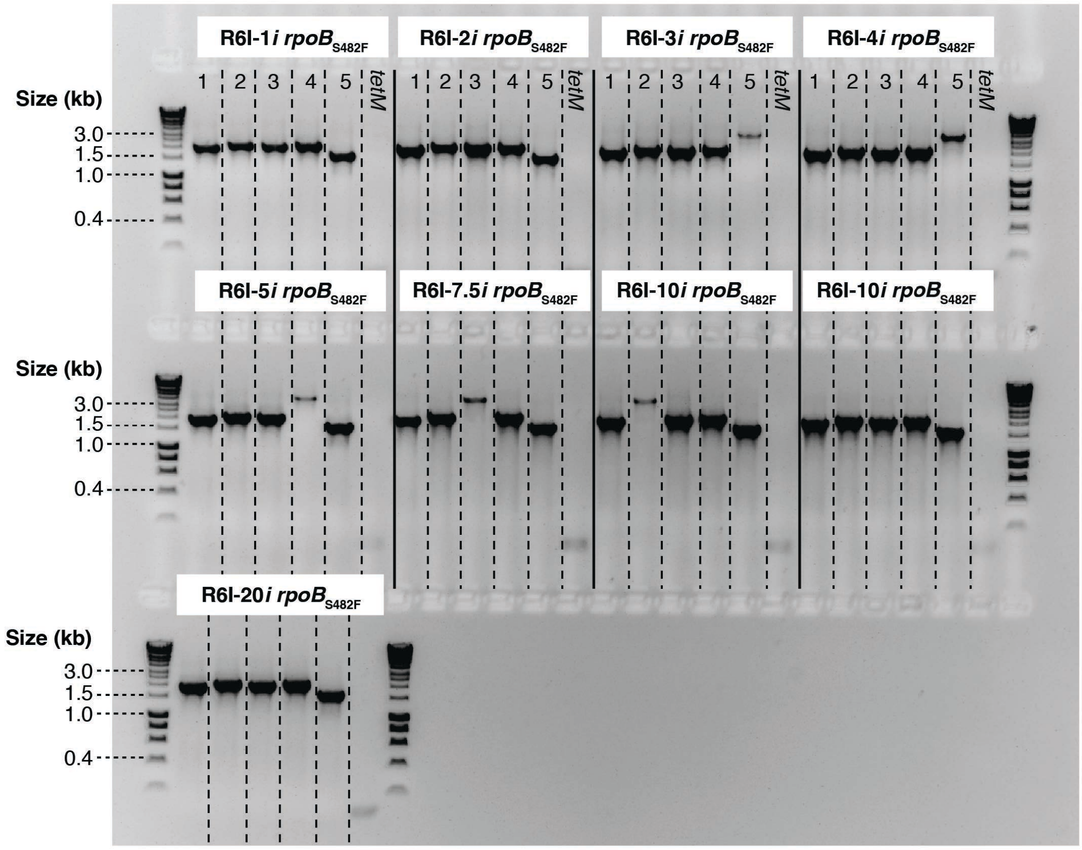

Figure S5 - Validating the construction of R6I-*Li* $rpoB_{S482F}$ genotypes. The R6I-*Li* strains were transformed with the PCR amplified rifampicin resistance allele of *rpoB* ($rpoB_{S482F}$) with primers rpoB.L and rpoB.R. Genomic DNA was extracted from these strains for the transformation experiments described in Fig 1. To verify no changes had occurred in the ICE*Sp*23FST81 region, validation PCRs using primer pairs insertLadder.1FR ('1'), insertLadder.2FR ('2'), insertLadder.3FR ('3'), insertLadder.4FR ('4'), insertLadder.5FR ('5'), and tetM.LR ('*tetM*') were run. Hyperladder 1kb was used as a DNA size marker on this 1% agarose gel. Each lane contains 5 µL of PCR product or Hyperladder 1kb. The PCR products correspond to the predicted sizes shown in Fig S2, confirming that no detectable changes had occurred to the ICE*Sp*23FST81 region of the strains.

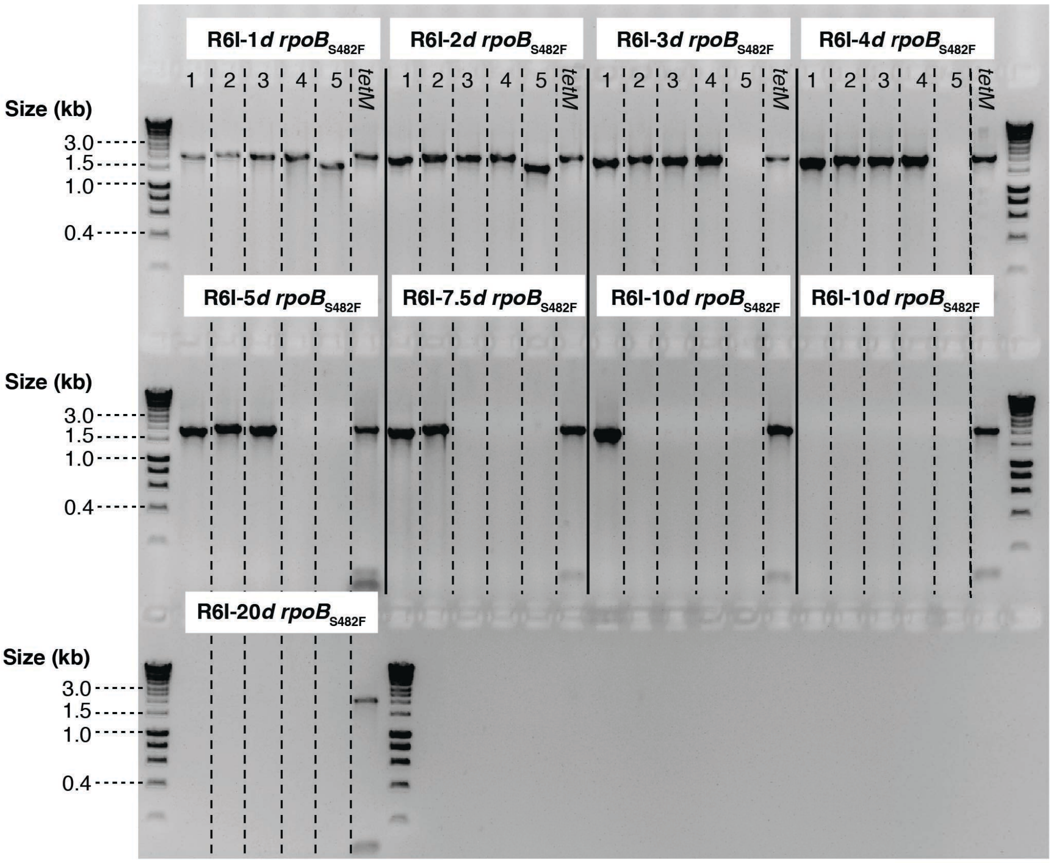

Figure S6 - Validating the construction of R6I-*Ld rpoB*$_{S482F}$ genotypes. The R6I-*Ld* strains were transformed with the PCR amplified rifampicin resistance-conferring allele of *rpoB* (*rpoB*$_{S482F}$) with primers rpoB.L and rpoB.R. Genomic DNA was extracted from these strains for the transformation experiments described in Fig 1. To verify no changes had occurred in the ICE*Sp*23FST81 region, validation PCRs using primers insertLadder.1FR ('1'), insertLadder.2FR ('2'), insertLadder.3FR ('3'), insertLadder.4FR ('4'), insertLadder.5FR ('5'), and tetM.LR ('*tetM'*) were run. Hyperladder 1kb was used as a DNA size marker on this 1% agarose gel. Each lane contains 5 μL of PCR product or Hyperladder 1kb. The PCR products correspond to the predicted sizes shown in Fig S2, confirming that no detectable changes had occurred to the ICE*Sp*23FST81 region of the strains.

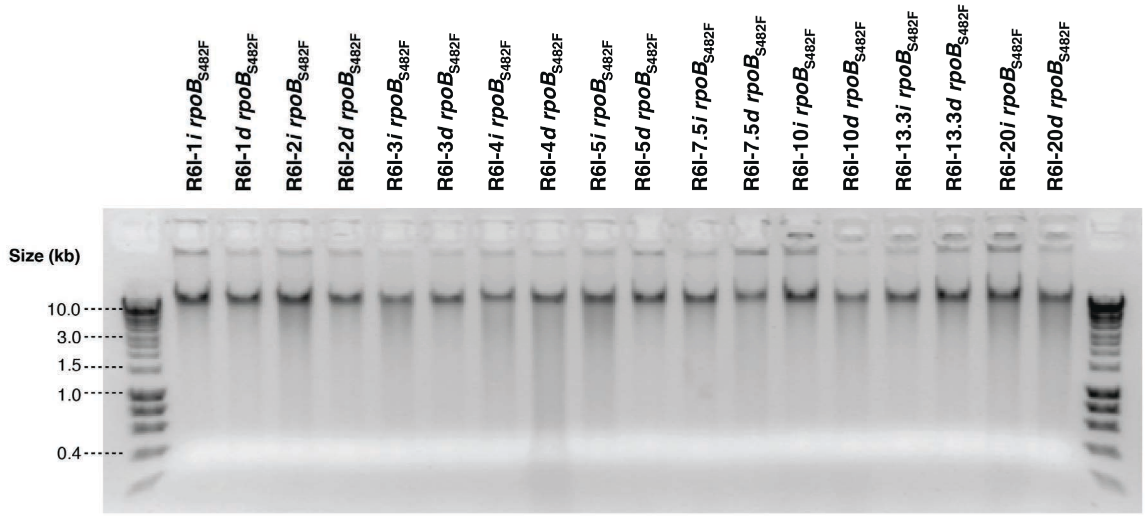

Figure S7 – Genomic DNA from R6I-*Li* and R6I-*Ld* genotypes used for transformation. Genomic DNA (5 µL) was visualised on 1% agarose gel relative to 5 µL Hyperladder 1kb used as a DNA size marker. This shows the concentrations of the genomic DNA samples after standardisation using electrophoresis and the NanoDrop 1000 spectrophotometer. The distribution of fragment sizes demonstrates the DNA had not undergone substantial degradation into small fragments prior to the experiments.

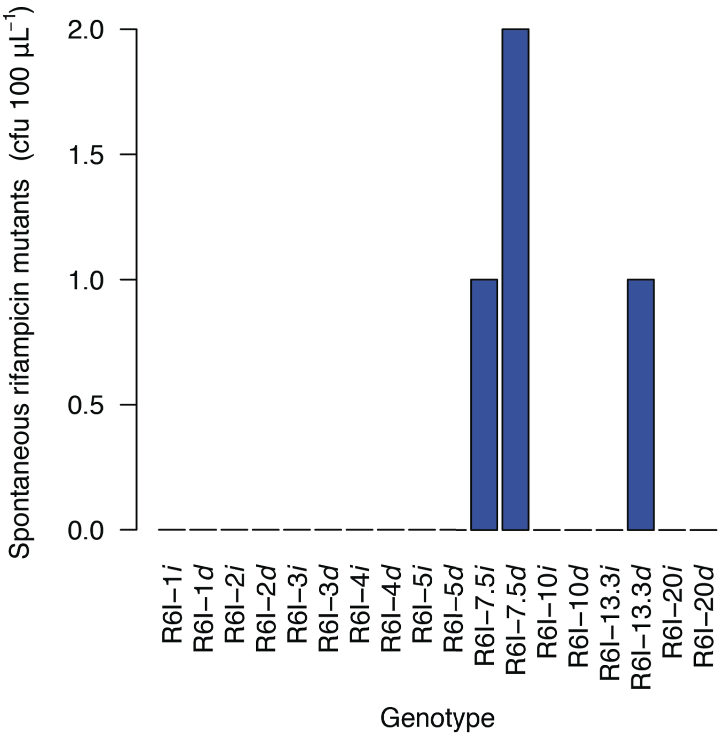

Figure S8 – Contribution of spontaneous mutation to rifampicin resistance. Each genotype was subject to a mock transformation, without donor DNA, and 100 µL plated on THY agar plates supplemented with rifampicin. Colonies were counted as a measure of the contribution of spontaneous mutation to the observed frequency of rifampicin-resistant colonies, used to infer $e\{S\}$. The absence of spontaneous mutants from most plates, and the low frequency in others, suggests this process has little effect on the results relative to the rate with which recipients were transformed with the *rpoB*$_{\text{S482F}}$ allele. Hence this process should not have much effect on estimates of $e\{S\}$, as this study only included experiments in which at least 250 rifampicin-resistant transformants per millilitre were estimated to be present.

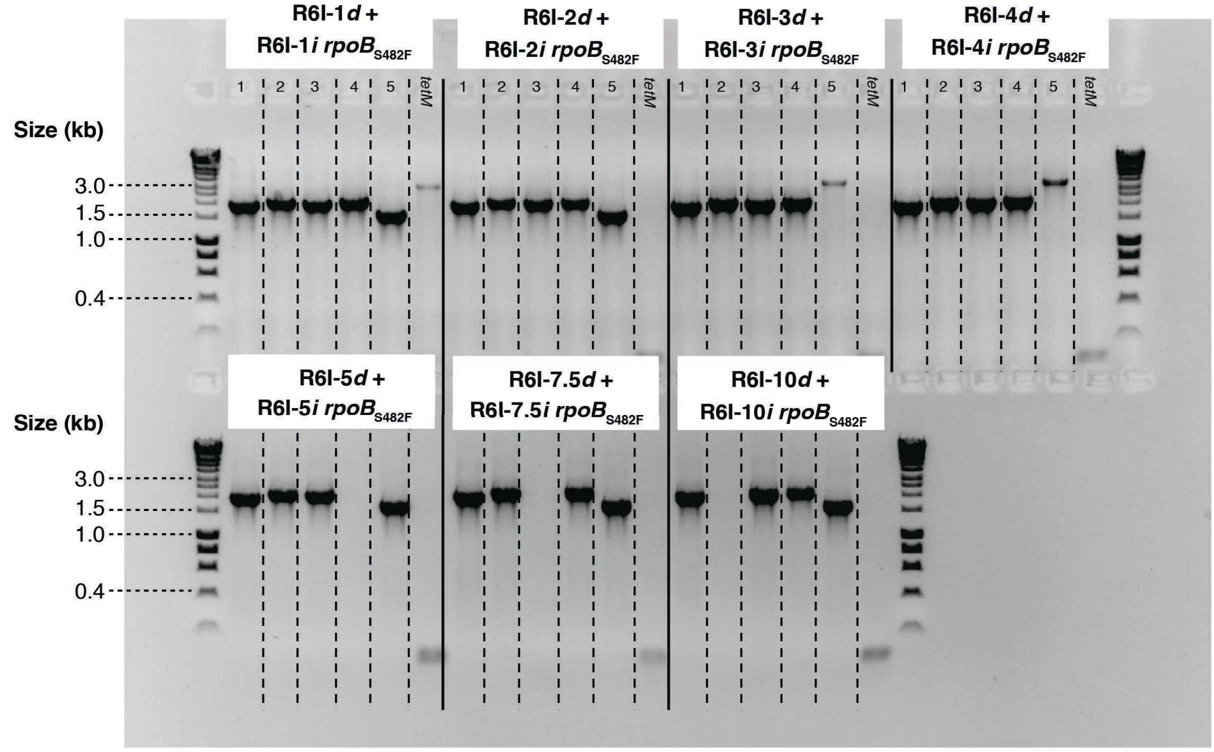

Figure S9 – Validating the specificity of erythromycin selection for acquisition of the *L* kb segments. R6I-*Ld* strains were transformed with genomic DNA extracted from the respective R6I-*Li* *rpoB*$_{S482F}$ strains, then subject to selection on THY agar plates containing 1 µg mL^{-1} erythromycin. To ensure this selection was specific for recombinations that inserted all the intervening DNA between the two halves of the *tetM* gene, all erythromycin-resistant colonies from a particular transformation were cultured in a single 10 mL THY broth culture, from which genomic DNA was extracted, and used as the template for validation PCRs (Fig S2). These reactions used primer pairs insertLadder.1FR ('1'), insertLadder.2FR ('2'), insertLadder.3FR ('3'), insertLadder.4FR ('4'), insertLadder.5FR ('5'), and tetM.LR ('*tetM*'). Hyperladder 1kb (Bioline) was used as a DNA size marker. Each lane contains 5 µL of PCR product or Hyperladder 1kb. The PCR products correspond to the predicted sizes for R6I-*Li* strains shown in Fig S2, with no false positive products from intact *tetM* genes found in the untransformed R6I-*Ld* recipient strains, confirming that erythromycin selection had high specificity for recombinants. In this experiment, no erythromycin-resistant colonies were observed following transformation of either R6I-13.3*d* or R6I-20*d*, therefore the no results for these values of *L* are shown.

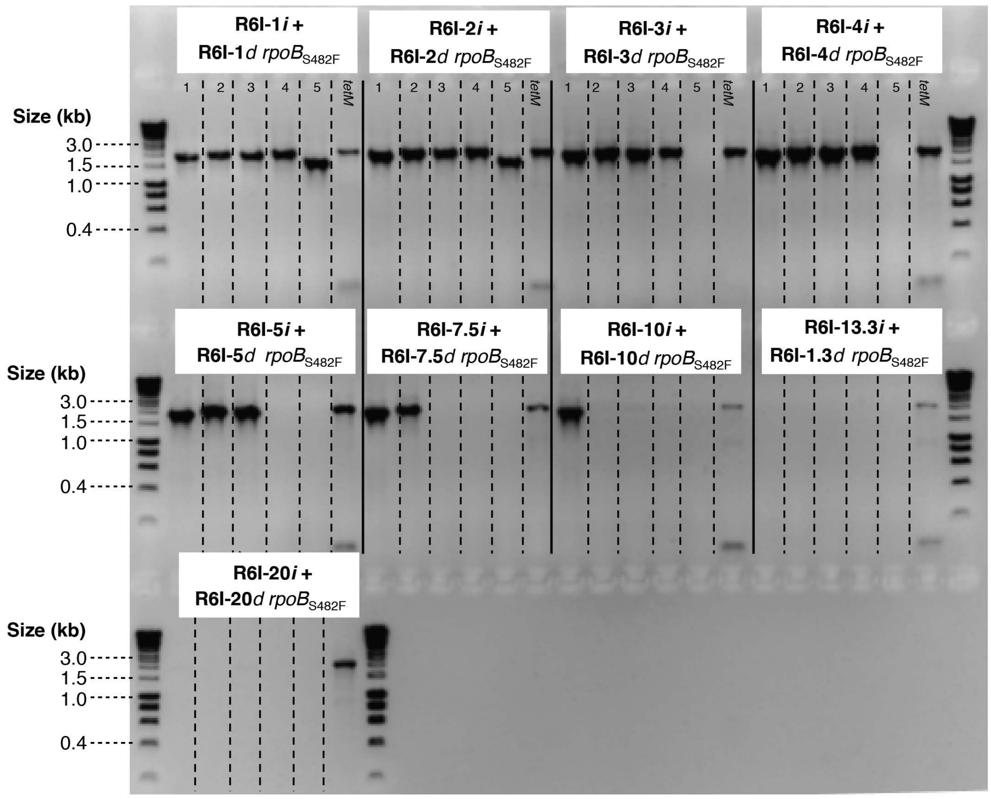

Figure S10 – Validating the specificity of tetracycline selection for removal of the *L* kb segments. R6I-*Li* strains were transformed with genomic DNA extracted from the respective R6I-*Ld* $rpoB_{S482F}$ strains, and then subject to selection on THY agar plates containing 10 μg mL^{-1} tetracycline. To ensure this selection was specific for recombinations that removed all the intervening DNA between the two halves of the *tetM* gene, all tetracycline-resistant colonies from a particular transformation were cultured in a single 10 mL THY broth culture, from which genomic DNA was extracted, and used as the template for validation PCRs (Fig S2). These reactions used primer pairs insertLadder.1FR ('1'), insertLadder.2FR ('2'), insertLadder.3FR ('3'), insertLadder.4FR ('4'), insertLadder.5FR ('5'), and tetM.LR ('*tetM*'). Hyperladder 1kb (Bioline) was used as a DNA size marker. Each lane contains 5 μL of PCR product or Hyperladder 1kb. The PCR products correspond to the predicted sizes shown in Fig S2 for R6I-*Ld* strains, with no false positive products from the DNA between the two *tetM* halves found in the untransformed recipient R6I-*Li* strains, confirming that tetracycline selection had high specificity for recombinants.

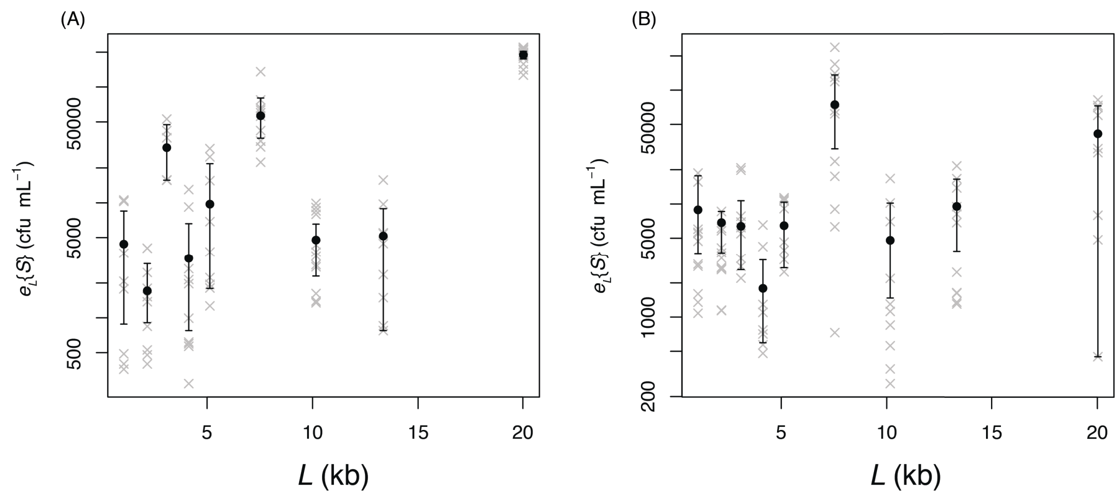

Figure S11 – Variation in transformation rates between genotypes. (A) Variation in transformability between R6I-*Li* genotypes. Each cross represents an estimated $e\{S\}$ measurement based on counts of rifampicin-resistant mutants from the experiments shown in Fig 2A. L denotes the length of heterology in the genotype. Each point represents the τ_g estimate from the model fit shown in Fig 2A. The error bars span the full range of τ_g estimates across the 100 bootstrap replicates. (B) Variation in transformability between R6I-*Ld* genotypes, shown as for panel (A), based on counts of rifampicin-resistant mutants from the experiments shown in Fig 2B.

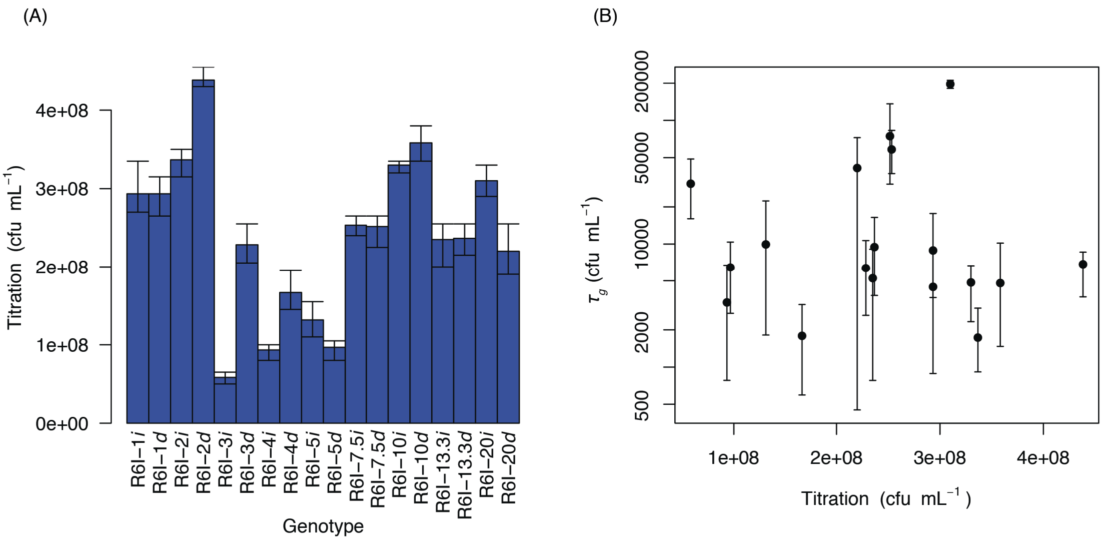

Figure S12 – Growth density of genotypes. Triplicate mock transformations, lacking donor DNA, were performed for each genotype, and the total cell density estimated by live cell count titration 3 h post-transformation. (A) Bar plot of cell densities. The bars shows the mean estimate, with an error bar indicating the range between maximum and minimum cell counts. (B) Relationship with genotype transformability. The total cell count was plotted against the τ_g estimates and associated uncertainty estimates in Fig S11. The lack of correlation demonstrates the variation in $e\{S\}$ is not explained by differences in growth rates between genotypes.

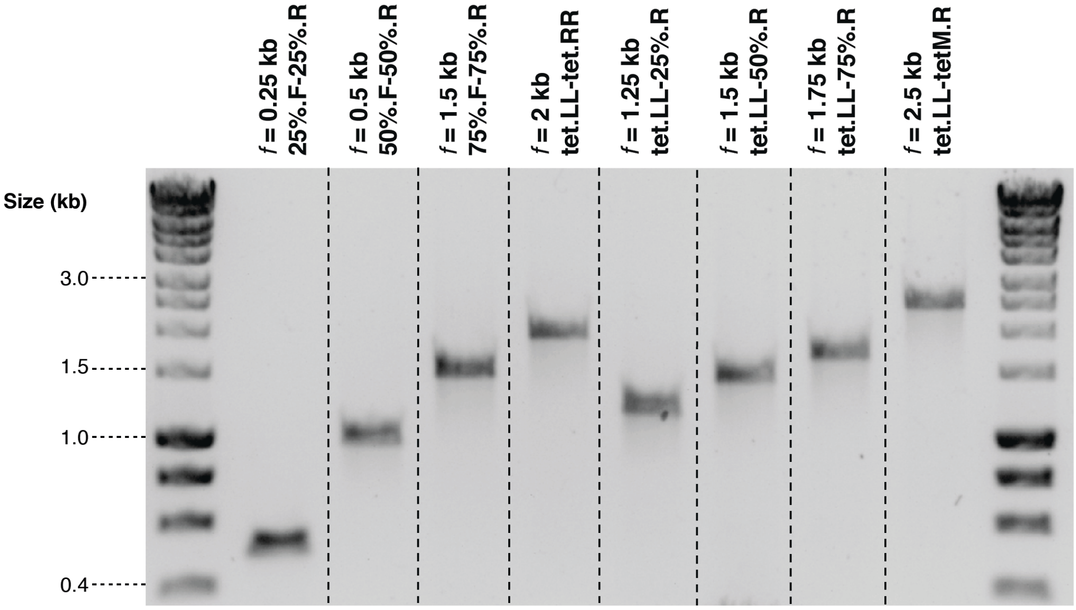

Figure S13 – DNA used to assay the properties of homologous arms required for deletion of regions of heterology. These lanes show the size, concentration and purity of the PCR amplicons from *tetM* used to remove *L* kb insertions from within the *tetM* gene, as shown in Fig 3. Each lane is labelled at the top with the fragment size, *f*, and the primer pair used to generate the amplicon (Table S2). Each of these was mixed with genomic DNA from a rifampicin-resistant *S. pneumoniae* TIGR4Δ*cps* mutant, which was added to each transformation shown in Fig 3 in order to standardise $e_D\{j\}$ rates by $e\{S\}$ rates.

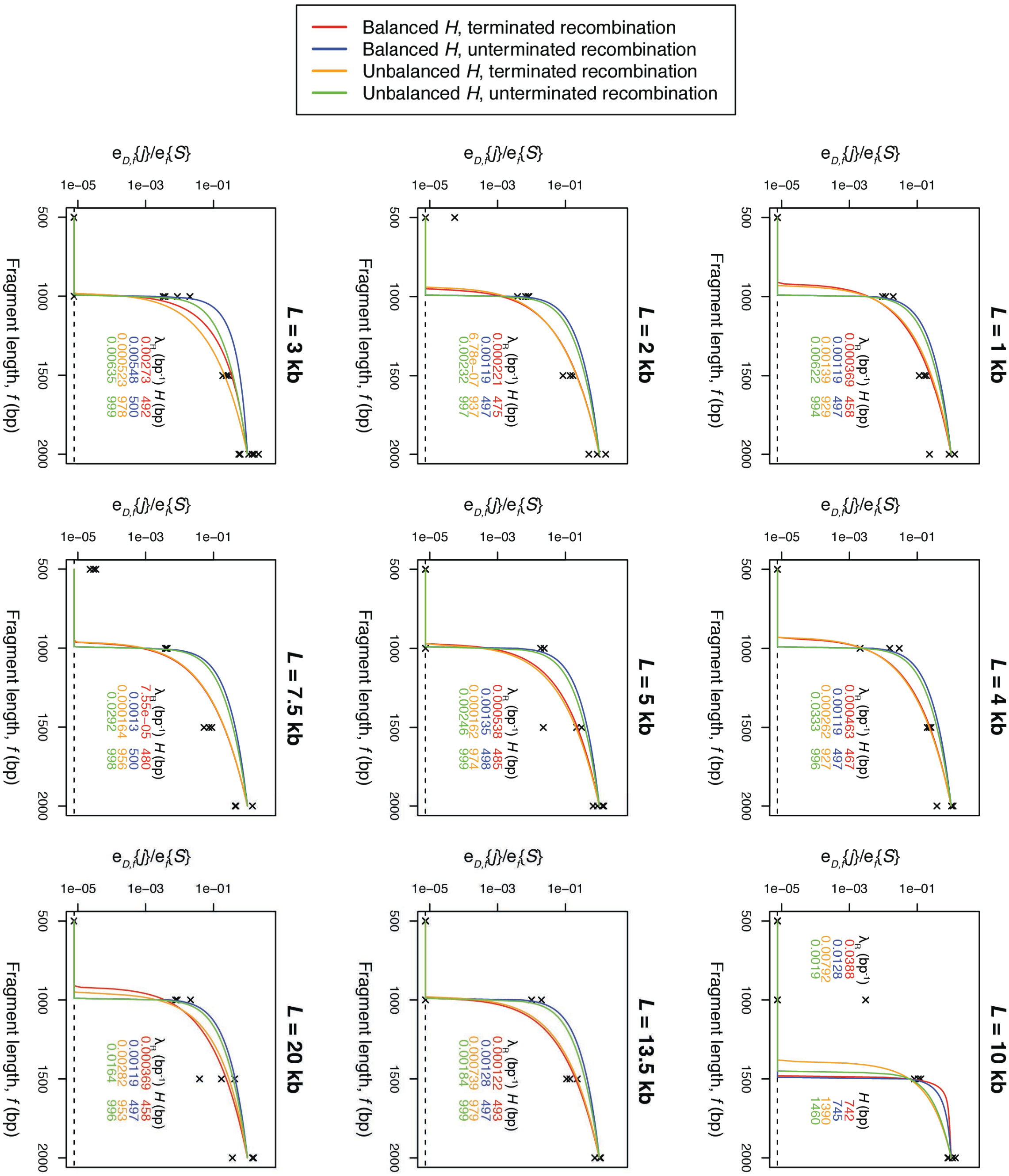

Figure S14 – Separate fits of the homologous arm length models to each R6I-*Li* genotype used as a recipient in the experiments included in Fig 3B. Each panel shows the least squares fit of the four models described in Text S2 to the three replicates for each of the R6I-*Li* genotypes. The estimated H and λ_R for each fit are shown on each plot, coloured according to the model structure, as indicated by the key.

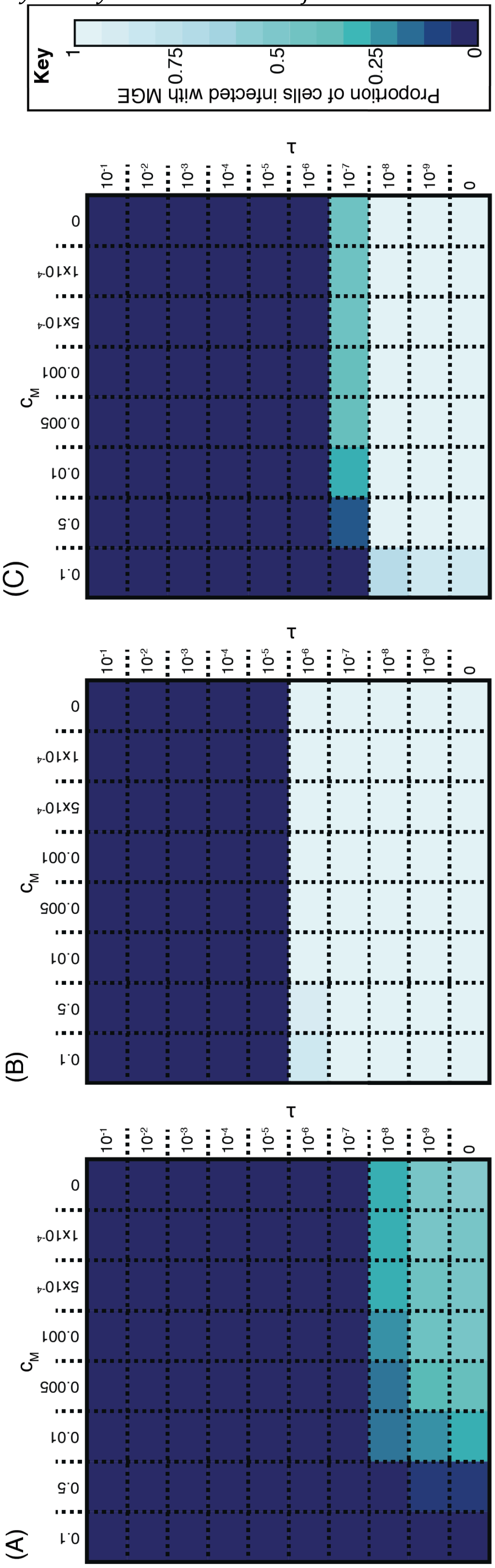
Key
Proportion of cells infected with MGE
1
0.75
0.5
0.25
0
(A)
(B)
(C)
τ
c_M
0
1x10⁻⁴
5x10⁻⁴
0.001
0.005
0.01
0.5
0.1
10⁻¹
10⁻²
10⁻³
10⁻⁴
10⁻⁵
10⁻⁶
10⁻⁷
10⁻⁸
10⁻⁹
0

Figure S15 - Simulating the conflict between mobile genetic elements (MGEs) and transformation with transformation asymmetries estimated from this work. Simulations were run using a previously described model (Croucher et al. 2016). Each parameter set was run in triplicate for 1,000 units of time, with 10^3 timesteps per unit of time. Simulations had the same growth rates ($\gamma = 0.2\ t^{-1}$), washout rate ($\omega = 0.6\ t^{-1}$) and carrying capacity ($\kappa = 10^6$) as previously, with no cell-cell killing or competence phase ($g_C = k_C = e_C = 0$). Each heatmap cell's colour represents the mean prevalence of the relevant MGE across three replicate simulations, according to the key. The horizontal axis shows variation in the fitness cost of the MGE to its host bacterium, c_M; where $c_M = 0$, the MGE is neutral. The vertical axis shows variation in the rate of transformation, τ; where $\tau = 0$, bacterial evolution is entirely clonal. (A) Simulating the evolutionary dynamics of a neutral or deleterious IS insertion, which was not autonomously transmissible ($\beta = 0$), did not kill its host cell on activation ($a = 0$) and was 1 kb in length ($\varphi_L = 0.29$). Transformation efficiently removed such IS elements at high τ, despite φ_L being close to one and many simulated insertions being near-neutral in terms of selective cost. (B) Simulating the evolutionary dynamics of a prophage, based on the 'more horizontal' MGE parameterised previously. Consequently, this MGE was highly transmissible ($\beta = 10^{-4}$, activation rate of $f = 0.05$, burst size of $b = 10$), killed its host cell on activation ($a = 1$) and was 30 kb in length ($\varphi_L = 1.2\text{x}10^{-5}$). Transformation is only effective at eliminating this MGE from the population at high τ, as this element rapidly transmits horizontally between cells, with little dependence on 'vertical' transmission, which can be inhibited by transformation. (C) Simulating the evolutionary dynamics of an integrative and conjugative element, based on the 'high β more vertical' MGE parameterised previously. This was infrequently transmitted with high efficiency ($\beta = 10^{-1}$, $b = 5$, $f = 0.005$), did not kill its host cell on activation ($a = 0$) and was 80 kb in length ($\varphi_L = 3.5\text{x}10^{-13}$). Such highly asymmetric transformation efficiently blocks the transmission of this MGE.

Supplementary Tables

Table S1 - Parameters from fitting models for homologous arm lengths to datasets stratified by *L*. Where *L* is specified as a number, the values refer to the fit to transformations of a particular R6I-*Li* genotype; where *L* is defined as 'All', it refers to the overall fit in Fig 3B; and where it is defined as '10 (unbalanced)', it refers to the experiments shown in Fig 3D.

Model	*L* (kb)	λ_R (bp^{-1})	*H* (bp)
Balanced H, terminated recombination	All	5.45E-05	469
Balanced H, unterminated recombination	All	1.83E-05	494
Unbalanced H, terminated recombination	All	2.78E-05	469
Unbalanced H, unterminated recombination	All	5.43E-02	499
Balanced H, terminated recombination	1	3.69E-04	458
Balanced H, unterminated recombination	1	1.19E-03	497
Unbalanced H, terminated recombination	1	1.39E-03	465
Unbalanced H, unterminated recombination	1	6.22E-03	498
Balanced H, terminated recombination	2	2.21E-04	476
Balanced H, unterminated recombination	2	1.19E-03	497
Unbalanced H, terminated recombination	2	6.78E-07	469
Unbalanced H, unterminated recombination	2	2.32E-03	499
Balanced H, terminated recombination	3	2.73E-03	492
Balanced H, unterminated recombination	3	5.48E-03	500
Unbalanced H, terminated recombination	3	5.23E-04	489
Unbalanced H, unterminated recombination	3	6.35E-03	500
Balanced H, terminated recombination	4	4.63E-04	467
Balanced H, unterminated recombination	4	1.19E-03	497
Unbalanced H, terminated recombination	4	2.62E-04	464
Unbalanced H, unterminated recombination	4	3.33E-02	498
Balanced H, terminated recombination	5	5.38E-04	486
Balanced H, unterminated recombination	5	1.35E-03	498

Unbalanced H, terminated recombination	5	1.62E-04	487
Unbalanced H, unterminated recombination	5	2.46E-03	500
Balanced H, terminated recombination	7.5	7.55E-05	481
Balanced H, unterminated recombination	7.5	1.30E-03	500
Unbalanced H, terminated recombination	7.5	1.64E-04	478
Unbalanced H, unterminated recombination	7.5	2.92E-02	500
Balanced H, terminated recombination	10	3.88E-02	743
Balanced H, unterminated recombination	10	1.28E-02	746
Unbalanced H, terminated recombination	10	7.92E-03	695
Unbalanced H, unterminated recombination	10	1.90E-03	730
Balanced H, terminated recombination	13.3	1.22E-04	493
Balanced H, unterminated recombination	13.3	1.28E-03	497
Unbalanced H, terminated recombination	13.3	7.39E-04	490
Unbalanced H, unterminated recombination	13.3	1.84E-03	500
Balanced H, terminated recombination	20	3.69E-04	458
Balanced H, unterminated recombination	20	1.19E-03	497
Unbalanced H, terminated recombination	20	2.82E-03	477
Unbalanced H, unterminated recombination	20	1.64E-02	498
Balanced H, terminated recombination	10 (unbalanced)	7.25E-05	250
Balanced H, unterminated recombination	10 (unbalanced)	1.12E-04	212
Unbalanced H, terminated recombination	10 (unbalanced)	3.19E-05	588
Unbalanced H, unterminated recombination	10 (unbalanced)	9.45E-03	623

Table S2 - Primers used in the procedures described in this study. The restriction enzyme cut sites are underlined in the sequences.

Primer name	Forward/Reverse	Sequence	5' restriction sites
ivr.LL	F	cgcaaggaagctggtattaca	-
ivr.CR.A	R	gtagggcccttccatcagcaaacttccca	*Apa*I
ivr.CR.B	R	gtaggatcccttccatcagcaaacttccca	*Bam*HI
ivr.CL	F	gtaggatcctgaaccctccgcattctaaaa	*Bam*HI
ivr.RR	R	caatctgaagacctagaaccttgct	-
Jns.F	F	ttgggcccccgtttgatttttaatggataatgtg	*Apa*I
Jns.R	R	atggatcccctttccttatgcttttggacg	*Bam*HI
tet.LL	F	cgacagccagtgaactttcc	-
tet.LLX	F	tatctagacgacagccagtgaactttcc	*Xba*I
tet.CRA	R	tagggcccgagtttgtgcttgtacgcca	*Apa*I
tet.CRB	R	tagggatccgagtttgtgcttgtacgcca	*Bam*HI
tet.CLB	F	gccggatcccatgcacttaggaaaatgggga	*Bam*HI
tet.RR	R	tgtactccgctccctaatggaa	-
2A	F	tagcggactgacacaatgga	-
2B	R	tagggcccaatcaaccgtcccctcactt	*Apa*I
3A	L	taggatccgaccgactacaagacaagaaca	-
3B	R	tatctagacaatcaaaagaagtagtcgggg	*Xba*I
4A	L	taggatccaccagcccagattccaacaa	*Bam*HI
4B	R	tatctagatttcatgaacagaagaagcaggc	*Xba*I
5A	L	tgcatttcgttccactgac	-
5B	R	tatctagaccacacggtcaataccgatac	*Xba*I
7.5A	L	taggatccaaagccctatctactgtccg	*Bam*HI
7.5B	R	tggtcggctatactggtact	-
8.5A	L	actttgggattcctgtggct	-
8.5B	R	ttctagatccagttctccgacaatgct	*Xba*I
10A	L	taggatccagcctattatgcagtccgtga	*Bam*HI

10B	R	tgagttcgccaatggaagt	-
11A	L	atccaacagcagacagtcca	-
11B	R	tatctagaacttctgcgtcttcaatggg	*Xba*I
17A	L	cctttcccagctccagatgt	-
17B	R	tatctagacccaaacaagcctatcgttcc	*Xba*I
18A	L	taggatccagctaatcagtttcacagct	*Bam*HI
18B	R	gtctatgcggtacaaggggt	-
kan.L	F	gtagggcccgtggtttcaaaatcggctcc	*Apa*I
kan.R	R	gtaggatccgggacccctatctagcgaac	*Bam*HI
tetM.L	F	tagggcccgttaataaatatgcggcaag	*Apa*I
tetM.R	R	taggatccctaagttattttattgaacatatatcg	*Bam*HI
ermB.L	F	tagggcccggcggaaacgtaaaagaag	*Apa*I
ermB.R	R	gccggatccgaattatttcctcccgttaaataatag	*Bam*HI
rpoB.L	F	cgygarcgbatgtcngtwca	-
rpoB.R	R	tcrtcngcwgtyarccaaac	-
insertLadder.1F	F	agtcgctaggtagaaaggagac	-
insertLadder.1R	R	gttcatgggagcaaaggac	-
insertLadder.2F	F	cgacaacagacgtacagcag	-
insertLadder.2R	R	tgaaagccacaggaatcc	-
insertLadder.3F	F	cgtgtcactctttgcagtg	-
insertLadder.3R	R	tggtcggctatactggtact	-
insertLadder.4F	F	gccattcgtgcagtaaccaa	-
insertLadder.4R	R	cgtccaagtttccgctgtag	-
insertLadder.5F	F	tgtgggaagtcgtgtgatga	-
insertLadder.5R	R	agtgggatatgctgggtcac	-
25%.F	F	cagaattaggaagcgtggac	-
25%.R	R	tgctttcctcttgttcgagt	-
50%.F	F	cctttatcatgtgattctaaagtatc	-
50%.R	R	tgtaatttttatttttccttttcc	-
75%.F	F	gaaaagaacgggagtaattggaag	-

Supplementary References

Croucher NJ, Coupland PG, Stevenson AE, Callendrello A, Bentley SD, Hanage WP. 2014. Diversification of bacterial genome content through distinct mechanisms over different timescales. Nat. Commun. 5:5471.

Croucher NJ, Harris SR, Barquist L, Parkhill J, Bentley SD. 2012. A high-resolution view of genome-wide pneumococcal transformation. PLoS Pathog 8:e1002745.

Croucher NJ, Harris SR, Fraser C, Quail MA, Burton J, van der Linden M, McGee L, von Gottberg A, Song JH, Ko KS, et al. 2011. Rapid pneumococcal evolution in response to clinical interventions. Science 331:430–434.

Croucher NJ, Mostowy R, Wymant C, Turner P, Bentley SD, Fraser C. 2016. Horizontal DNA Transfer Mechanisms of Bacteria as Weapons of Intragenomic Conflict. PLOS Biol. 14:e1002394.

Croucher NJ, Walker D, Romero P, Lennard N, Paterson GK, Bason NC, Mitchell AM, Quail MA, Andrew PW, Parkhill J, et al. 2009. Role of conjugative elements in the evolution of the multidrug-resistant pandemic clone *Streptococcus pneumoniae*$^{\mathrm{Spain23F}}$ ST81. J Bacteriol 191:1480–1489.

Henningsen A, Toomet O. 2011. MaxLik: A package for maximum likelihood estimation in R. Comput. Stat. 26:443–458.

Pearce BJ, Iannelli F, Pozzi G. 2002. Construction of new unencapsulated (rough) strains of *Streptococcus pneumoniae*. Res. Microbiol. 153:243–247.

Shoemaker NB, Smith MD, Guild WR. 1980. DNase-resistant transfer of chromosomal *cat* and *tet* insertions by filter mating in Pneumococcus. Plasmid 3:80–87.

Sung CK, Li H, Claverys JP, Morrison DA. 2001. An *rpsL* cassette, Janus, for gene replacement through negative selection in *Streptococcus pneumoniae*. Appl. Environ. Microbiol. 67:5190–5196.

Tiraby JG, Fox MS. 1973. Marker discrimination in transformation and mutation of pneumococcus. Proc. Natl. Acad. Sci. U. S. A. 70:3541–3545.
